# Aging Effects on Metabolic Sensor and Glycogen Metabolism in Old Male versus Female Rat Primary Hypothalamic Astrocyte Cultures

**DOI:** 10.3390/neuroglia6040041

**Published:** 2025-11-01

**Authors:** Rami Shrestha, Madhu Babu Pasula, Karen P. Briski

**Affiliations:** School of Basic Pharmaceutical and Toxicological Sciences, College of Pharmacy, University of Louisiana Monroe, Monroe, LA 71201

**Keywords:** GLUT2 siRNA, AMPK, glycogen phosphorylase, LC-MS, glycogen

## Abstract

**Background/Objectives:**

Compartmentalized glucose metabolism in the brain contributes to neuro-metabolic stability and shapes hypothalamic control of glucose homeostasis. Glucose transporter-2 (GLUT2) is a plasma membrane glucose sensor that exerts sex-specific control of hypothalamic astrocyte glucose and glycogen metabolism. Aging causes counterregulatory dysfunction.

**Methods:**

Current research used Western blot and HPLC-electrospray ionization-mass spectrometry to investigate whether aging affects GLUT2-dependent hypothalamic astrocyte metabolic sensor and glycogen enzyme protein expression and glycogen mass according to sex.

**Results:**

Data document GLUT2-dependent up-regulated glucokinase (GCK) protein in glucose-deprived old male and female astrocyte cultures, unlike GLUT2 inhibition of this protein in young astrocytes. Glucoprivation of old male and female astrocytes caused GLUT2-independent down-regulation of 5’-AMP-activated protein kinase (AMPK) protein, indicating loss of GLUT2 stimulation of this protein with age. This metabolic stress also caused GLUT2-dependent suppression of phospho-AMPK profiles in each sex, differing from GLUT2-mediated glucoprivic enhancement of activated AMPK in young male astrocytes and phospho-AMPK insensitivity to glucoprivation in young female cultures. GS and GP isoform proteins were refractory to glucoprivation of old male cultures, contrary to down-regulation of these proteins in young glucose-deprived male astrocytes. Aging elicited a shift from GLUT2 inhibition to stimulation of male astrocyte glycogen accumulation and caused gain of GLUT2 control of female astrocyte glycogen.

**Conclusions:**

Outcomes document sex-specific, aging-related alterations in GLUT2 control of hypothalamic astrocyte glucose and ATP monitoring and glycogen mass and metabolism. Results warrant future initiatives to assess how these adjustments in hypothalamic astrocyte function may affect neural operations that are shaped by astrocyte-neuron metabolic partnership.

## 1. Introduction

The brain operates at the apex of glucose consumption in the body owing to high energy requirements of vital nerve cell functions, such as transmembrane electrolyte gradient preservation and synaptic discharge. Reductions in systemic glucose concentrations, exemplified by clinical hypoglycemia, pose a risk for neurological dysfunction or damage or demise of vulnerable nerve cell populations. The brain incorporates a dedicated nerve cell network that maintains tight control of body-wide glucose homeostasis [1–3]. This neural circuitry encompasses several interconnected structures located in multiple major brain regions including the hypothalamus; these loci operate as an integrated system to regulate appropriate counteractive autonomic, neuroendocrine, and behavioral motor responses to diminished systemic glucose availability [4–6].

Astrocytes are an important structural and functional component of brain cytoarchitecture. Cytoplasmic extensions of these neuroglia encircle brain capillaries, where they contact capillary endothelial cells to create the semi-permeable blood-brain barrier [7–9]. In that location, astrocytes control brain uptake of glucose and other critical ions and molecules. Astrocytes actively contribute to neuro-metabolic homeostasis [10]. Inside the astrocyte interior, glucose is either diverted to the glycogen reserve or catabolized via glycolysis to generate the oxidizable monocarboxylate L-lactate [11]. Cell type-specific monocarboxylate transporters transfer lactate from astrocytes to neurons to fuel nerve cell mitochondrial energy production [12]. Hypothalamic astrocyte-neuron metabolic coupling influences glucose counterregulatory outflow [13,14].

Glucoregulatory circuit function is shaped by input from chemo-sensors that monitor cellular supply of glucose or the energy currency ATP. Astrocyte plasma membrane glucose uptake is monitored by glucose transporter-2 (GLUT-2), a major facilitator transporter superfamily SLC2 gene-encoded protein [15–17]. GLUT2 is different from other GLUT family proteins as it exhibits low affinity for glucose, e.g. Km = 17 mM, which infers that glucose transport is unreservedly proportionate over a scale of relevant physiological concentrations [18]. This glucose transporter is a critical source of astrocyte input to the glucoregulatory network as neuroglucopenia-stimulated counterregulatory hormone release is reliant upon GLUT2 function in this distinctive brain cell compartment [19]. Intracellular astrocyte glucose levels are tracked at the rate-limiting initial step of the glycolytic pathway by the unique hexokinase glucokinase/hexokinase IV (GCK), which facilitates phosphate transfer from ATP to glucose to yield glucose-6-phosphate [20–27]. Glucokinase regulatory protein (GKRP) governs cytoplasmic GCK glucose binding [27]. In reaction to diminished cytoplasmic glucose levels, GKRP forms complexes with glucose-free GCK, which translocate from the cytoplasm to the nucleus to sequester inactive GCK. GKRP expression is a plausible indicator of brain cell metabolic sensitivity, as GKRP-expressing hindbrain A2 noradrenergic neurons exhibit neurotransmitter biosynthetic enzyme, GCK, and K_ATP_ channel sulfonylurea receptor-1 subunit transcriptional reactivity to hypoglycemia, yet these gene profiles are hypoglycemia-insensitive in A2 nerve cells lacking GKRP protein [28].

Cellular ATP content is monitored by the highly-sensitive, evolutionary ATP gauge 5’-AMP-activated protein kinase (AMPK). AMPK is actuated by phosphorylation at threonine-172 upon reductions in the intracellular AMP/ATP ratio [29–34]. AMPK renders cellular energy balance more positive by initiating operations that facilitate energy production, i.e. lipid oxidation and glucose uptake, while inhibiting activities that utilize energy, such as macromolecule production. AMPK is a heterotrimeric complex of catalytic (alpha) and regulatory (beta, gamma) subunits. AMPK catalytic subunits alpha-1 (PRKAA1/AMPKα1) and alpha-2 (PRKAA2/AMPKα2) are triggered to an equivalent magnitude when intracellular AMP levels increase [35]. There is ample evidence that GCK and AMPK operate in the hypothalamus to supply vital sensory cues to neural circuitries that control bodily energy and glucose homeostasis [36–44].

VMN astrocyte glycogen metabolism is a crucial metabolic variable that affects counterregulatory endocrine outflow [45]. Astrocyte glycogen is dynamically remodeled during metabolic homeostasis by passage of glucose through the glycogen shunt and provides needed lactate equivalents when neurological activity is elevated or during hypoglycemia [10]. Glycogen metabolism is governed by antagonistic glycogen synthase (GS) and glycogen phosphorylase (GP) actions, which catalyze glycogen accumulation or disassembly. GP is converted from non-active to active conformation by phosphorylation at Ser-14 and/or allosteric activation by AMP. GP-muscle type (GPmm) and GP-brain type (GPbb) enzyme variants are expressed in brain [46]. Our studies document the co-presence of these GP isoforms in hypothalamic astrocytes [47]. GPmm and GPbb exhibit significant differences in activation by the above effectors, as phosphorylation causes complete or partial activation of GPmm versus GPbb, yet AMP more potently activates GPbb relative to GPmm and is requisite for maximal GPbb Km and function [46]. Reports that GPbb and GPmm respectively achieve glucoprivic or noradrenergic stimulation of astrocyte glycogen glycogenolysis [48] support the notion that these variant enzymes may confer physiological stimulus-specific regulation of glycogen mobilization in the brain.

Aging has wide-ranging effects on somatic and homeostatic functions in the body, including neural regulation of metabolism. The elderly face the prospect of heightened risk of insulin-induced hypoglycemia (IIH)-associated brain injury as counteractive hormone outflow and neurogenic awareness are impaired with age [49–54]. There is a pressing need to ascertain the mechanism(s) that elicits aging-related counterregulatory dysfunction. Age-related impairment of astrocyte-nerve metabolic coupling is acknowledged [55], but insight on if and how aging may affect hypothalamic astrocyte glucose handling and glycogen amassment and mobilization in each sex remains limited. Recent studies using young adult rat primary hypothalamic astrocyte cultures show that GLUT2 enacts sex-specific control of hypothalamic astrocyte GCK and total/activated AMPK protein profiles and glycogen amassment [56]. Current research addressed the hypothesis that aging may cause sex-dimorphic adjustments in GLUT2-dependent metabolic sensor function and glycogen metabolism in old rats of one or both sexes during glucose sufficiency and/or deficiency.

## 2. Materials and Materials:

### 2.1. Primary astrocyte cell cultures

High purity astrocyte cultures were derived from hypothalamic tissue collected from young adult (2–3 months of age) or old (11–12 months of age) male and female Sprague Dawley albino rats (Rattus norvegicus), as described [57]. Protocols for animal handling and use followed NIH Guide for Care and Use of Laboratory Animals, 8^th^ Edition, and ARRIVE guidelines, and were implemented under approval by the ULM Institutional Animal Care and Use Committee [21KPB-01]. Briefly, tissue encompassing the entire hypothalamus was removed from each brain, using the following boundaries: optic chiasm rostral border (anterior); mammillary bodies rostral margin (posterior); lateral extent of tuber cinereum (lateral); top of the third ventricle (dorsal) as boundaries [56]. A single-cell suspension was prepared from each tissue block by pipet-dissociation in the presence of trypsin. For each astrocyte collection, suspended cells from three hypothalami dissected from animals of the same sex and age were combined in Dulbecco’s modified eagle medium (DMEM) high-glucose media (prod. no. 12800–017; ThermoFisherScientific, Waltham, MA, USA) containing 10.0% heat-inactivated fetal bovine serum (FBS) (GE Healthcare Bio-Sciences, Pittsburgh, PA, USA) and 1.0% penicillin–streptomycin (prod. no. 15140–122; ThermoFisherSci.). Three independent astrocyte collections were made for young and old rats of each sex to perform triplicate stand-alone experiments; thus n= 9 old male, n=9 old female; n=9 young male; and n=9 young female rats were used in current studies. Dissociated cells were incubated (37ᵒC; 5.0% CO_2_) in poly-D-lysine (prod. no. A-003-E; MilliporeSigma, Burlington, MA, USA)-coated T75 culture flasks containing plating media for fourteen days before elimination of microglia and oligodendrocytes [57]. Purified astrocytes were plated on poly-D-lysine-coated culture dishes, 1 × 10^6^ cells/100 mm^2^ cell density, prior to experimentation. Astrocyte culture homogeneity exceeding 95% was verified by analysis of the astrocyte marker protein glial fibrillary acidic protein (GFAP) by immunofluorescence staining and immunoblotting [57] [Supplementary Figure 1].

### 2.2. Experimental Design

After reaching 70% confluency, astrocyte cultures were incubated for 18 hours in high-glucose DMEM medium supplemented with 5.0% charcoal-stripped FBS (prod. no. 12676029; ThermoFisherSci.). Old astrocytes of each sex were exposed (72 hr) to high-glucose DMEM media containing Accell^™^ rat GLUT2 siRNA (prod. no. A-099803–14-0010, 5.0 nM; Horizon Discovery LTD, Lafayette, CO, USA) or Accell^™^ control non-targeting pool (scramble; SCR) siRNA (prod. no. D-001910–10-20, 5.0 nM; Horizon Disc.) in siRNA buffer (prod. no. B-002000-UB-100; Horizon Disc.) [Pasula et al., 2022]. This siRNA gene knockdown treatment causes an approximate 50% reduction in target gene product expression [Pasula et al., 2022]. SCR or GLUT2 siRNA-pretreated old rat astrocytes were then incubated (8 hr) in media containing (5.5 mM; G5.5) or lacking (0 mM; G0) of glucose, as described [Ibrahim et al., 2020]). Cells were then detached and suspended in lysis buffer (2.0% sodium dodecyl sulfate, 5% β-mercaptoethanol, 1 mM sodium orthovanadate, 10.0% glycerol, 60 mM Tris-HCl, pH 6.8% Young rat astrocyte control cultures were pretreated with SCR siRNA before incubation with media containing 5.5 mM glucose.

### 2.3. Western Blot Analysis

Astrocyte cell pellets were centrifuged, sonicated, and heat-denatured before dilution with Laemmli buffer. Sample lysate protein concentrations were analyzed by NanoDrop spectrophotometry (prod. no. ND-ONE-W, ThermoFisherSci.). Sample aliquots of comparable protein content were separated in Bio-Rad Stain Free 10% acrylamide gels (prod. no. 161–0183; Bio-Rad, Hercules, CA, USA). Stain-Free imaging technology for total protein quantification was used as loading control. After UV gel activation, proteins were transblotted to 0.45-μm PVDF-Plus membranes (prod. no. 1212639; Data Support Co., Panorama City, CA, USA) for automated Freedom RockerTM Blotbot^®^ (Next Advance, Inc., Troy, NY, USA) wash and antibody incubation processing. Target proteins were analyzed using triplicate independent astrocyte cultures. Membranes were blocked with Tris-buffered saline (TBS), pH 7.4, containing 2% bovine serum albumin (prod. no. 9048–46-8; VWR) and 0.2% Tween-20 (prod. no. 9005– 64-5; VWR), before incubation with primary antisera against GLUT2 (prod. no. PA5–97263, ThermoFisherSci.; RRID: AB_2809065; 1:1000), AMPKα1/2 (AMPK) (prod. no. 2532S, Cell Signaling Technology, Danvers, MA, USA; RRID: AB-330331; 1:2000), phosphoAMPKα1/2-Thr 172 (pAMPK; prod. no. 2535S; 1:1600; Cell Signal. Technol.; RRID: AB_331250; 1,2000), GCK (prod. no. bs-1796R; Bioss Antibodies, Inc., Woburn, MA, USA; RRID: AB_11095614; 1:1500), GKRP (prod. no. NBP2–03396, Novus Biologicals, Littleton, CO, USA; 1:1500), GPbb (prod. no. NBP1–32799, Novus Biol.; RRID: AB-2253353; 1:2,000), GPmm (prod. no. NBP2–16689; Novus Biol.1 2,000) or GS (prod. no. 3893S; Cell Signal. Technol.; RRID: AB_2279563; 1: 2,000) [Pasula et al., 2022]. Membranes were next treated with goat anti-rabbit (product number NEF812001EA, PerkinElmer, Waltham, MA, USA; 1:4,000) or anti-mouse (prod. no. NEF822001EA, PerkinElmer; 1:4000) peroxidase-labeled secondary antibodies, then exposed to SuperSignal West Femto chemiluminescent substrate (prod. no. 34096; ThermoFishSci.). Quantified Western blot target protein optical densities (O.D.) were normalized to total in-lane protein using a Bio-Rad ChemiDoc^™^ Touch Imaging System and Image Lab^™^ 6.0.0 software. A proprietary, intrinsically non-fluorescent trihalo compound in Bio-Rad Stain-Free gels is photo-activated upon UV exposure, thereby rendering all in-gel proteins fluorescent for summation of those O.D. measures. Software totals all protein measures in a single lane and relates that to target protein O.D. in that lane to generate a normalized O.D. value. Precision Plus protein molecular weight dual-color standards (prod. no. 161–0374; Bio-Rad,) were utilized as reference markers in every Western blot analysis.

### 2.4. Astrocyte LC-MS Glycogen Analysis

Astrocyte glycogen concentrations were measured as reported [58]. D-(+)-Glucose-1-phenyl-3-methyl-5-pyrazolone (PMP) was resolved using a Shodex Asahipak NH2P-40 3E column with acetonitrile:10 mM ammonium acetate (75:25 v/v; 0.2 mL/min) as mobile phase. D-(+)-Glucose-PMP ion chromatograms were extracted from Total Ion Current (TIC) at m/z 510.2 to acquire area-under-the curve data.

### 2.5. Statistical Analyses

For each sex, mean normalized target protein O.D. data were compared between young versus old control SCR siRNA/G5.5 treatment groups by *t* test. Protein O.D. measures for old male or female rat astrocyte treatment groups were analyzed by two-way analysis of variance and Student-Newman-Keuls *post-hoc* test. Astrocyte glycogen concentrations were analyzed among young or old astrocyte treatment groups by three-way analysis of variance and Student-Newman-Keuls *post-hoc* test. Mean differences were considered significant at *p*<0.05. Statistical differences between paired treatment groups are denoted by the following symbols: *p<0.05, **p<0.01, ***p<0.001.

## 3. Results

Brain glucose metabolism involves multiple cell compartments. Astrocytes engage in uptake, storage, and catabolism of glucose and transfer the oxidizable glycolytic product L-lactate to fuel nerve cell energy production. Hypothalamic astrocyte-neuron metabolic coupling shapes neural control of systemic glucose counterregulation. Aging is associated with counterregulatory dysfunction. Current research used old rat hypothalamic primary astrocyte cultures to address the premise that aging cause sex-dimorphic adjustments in GLUT2-dependent glucose handling and glycogen metabolism in these neuroglia.

**Figure 1.**
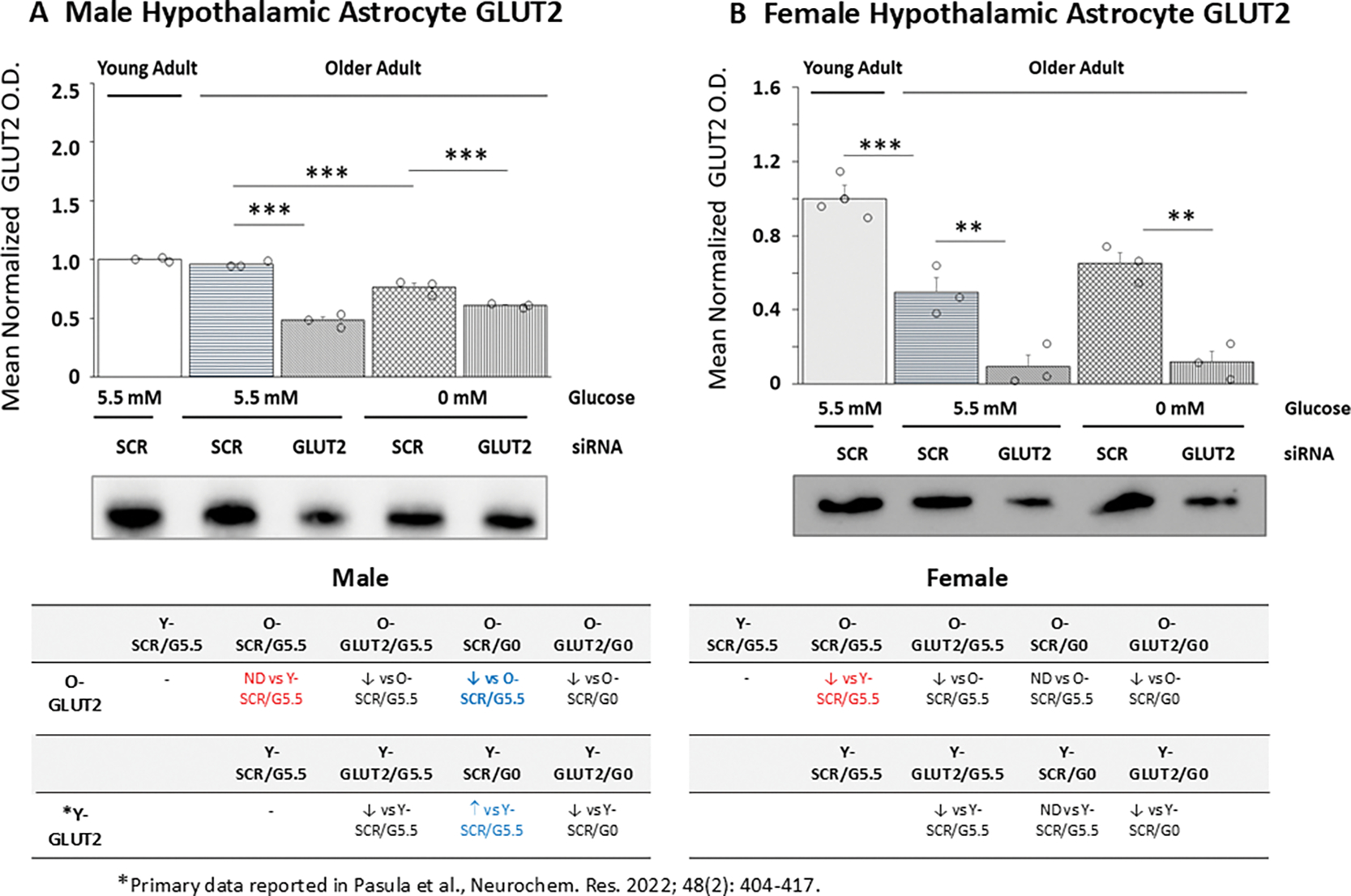
Effects of Glucose Transporter-2 (GLUT2) Gene Knockdown on Old Male and Female Hypothalamic Primary Astrocyte GLUT2 Protein Expression. Old male and female rat astrocyte cultures were pretreated with scramble (SCR) or GLUT2 siRNA prior to incubation in 5.5 (G5.5) or 0 (G0) mM glucose-containing media. Young adult male or female SCR siRNA/G5.5 astrocyte cultures served as controls. Astrocyte lysates were analyzed across treatment groups by Western blot for GLUT2 protein content in three independent experiments. Target protein optical density (O.D.) measures acquired in a Bio-Rad ChemiDoc^™^ Touch Imaging System were normalized to total in-lane protein (loading control) using Stain-Free technology and Bio-Rad Image Lab^™^ 6.0.0 software. Data depict mean normalized GLUT2 protein O.D. values ± S.E.M. for male (Figure 1A) and female (Figure 1B) astrocyte treatment groups. In each figure, the solid bar *at left* depicts mean GLUT2 O.D. for young adult astrocyte SCR siRNA/G5.5 cultures, whereas old male or female astrocyte treatment groups are illustrated as follows: SCR siRNA/G5.5 (horizontal-striped bars); GLUT2 siRNA/G5.5. (diagonal-striped bars); SCR siRNA/G0 (crosshatched bars); GLUT2 siRNA/G0 (vertical-striped bars). For each sex, mean normalized GLUT O.D. data were compared between young and old SCR siRNA/G5.5 groups by *t* test and old astrocyte treatment groups were analyzed by two-way ANOVA and Student-Newman-Keuls *post-hoc*, using GraphPad Prism, Vol. 8 software. Statistical differences between treatment group pairs are indicated by the following symbols: *p < 0.05; **p < 0.01; ***p < 0.001. Tables below Figure 1A and 1B summarize results shown in graphic format; results from prior studies involving SCR siRNA/G5.5, GLUT2 siRNA/G5.5, SCR siRNA/G0, or GLUT2 siRNA/G0 treatment on young adult male or female astrocyte GLUT2 protein expression are presented for comparison. Red font denotes a sex difference in age effect on SCR siRNA/G5.5 control group GLUT2 protein expression. Blue font indicates an age-related change in treatment effect on GLUT protein profiles.

Figure 1 illustrates effects of GLUT2 gene knockdown on GLUT2 protein expression in old male (Figure 1A) and female (Figure 1B) hypothalamic primary astrocyte cultures. Data in Figure 1A show that mean normalized GLUT2 O.D. values for young (solid white bar) versus old male (horizontal-striped, white bar) SCR siRNA/G5.5 control astrocyte cultures were equivalent [*p* = 0.103]. Old female astrocytes (gray horizontal-striped bar) exhibited diminished GLUT2 protein relative to young cell cultures (solid gray bar) (Figure 1B; *p*< 0.05). Each figure also depicts effects of GLUT2 gene knockdown [GLUT2 siRNA/G5.5; white (male) and gray (female) horizontal-striped bars], glucose deprivation [SCR siRNA/G0; white (male) and female (gray) cross-hatched bars]; or combinatory GLUT2 siRNA/glucoprivation treatment [GLUT2 siRNA/G0; white (male) and female (gray) vertical-striped bars] on GLUT2 protein in old male and female astrocyte cultures. Results indicate that GLUT2 siRNA administration significantly down-regulated GLUT2 protein expression in glucose-supplied or -deprived astrocytes of each sex. Glucose deprivation inhibited GLUT2 expression in old male rat astrocytes (SCR siRNA/G0 versus SCR siRNA/G5.5) but did not affect this protein profile in old female rat cultures. Images of uncropped Western blots presented in this and subsequent figures are shown in Supplementary Figures 2 and 3. Tables located below Figures 1A (male, *at lower left*) and 1B (female, *at lower right*) compare, for each sex, effects of GLUT2 siRNA or glucoprivation alone or in combination on GLUT2 protein expression in old (rows 1 and 2) versus young (rows 3 and 4) astrocyte cultures. Outcomes reveal that male astrocytes exhibit a switch in direction of GLUT2 protein response to glucose withdrawal (illustrated in blue font) because of aging.

**Figure 2.**
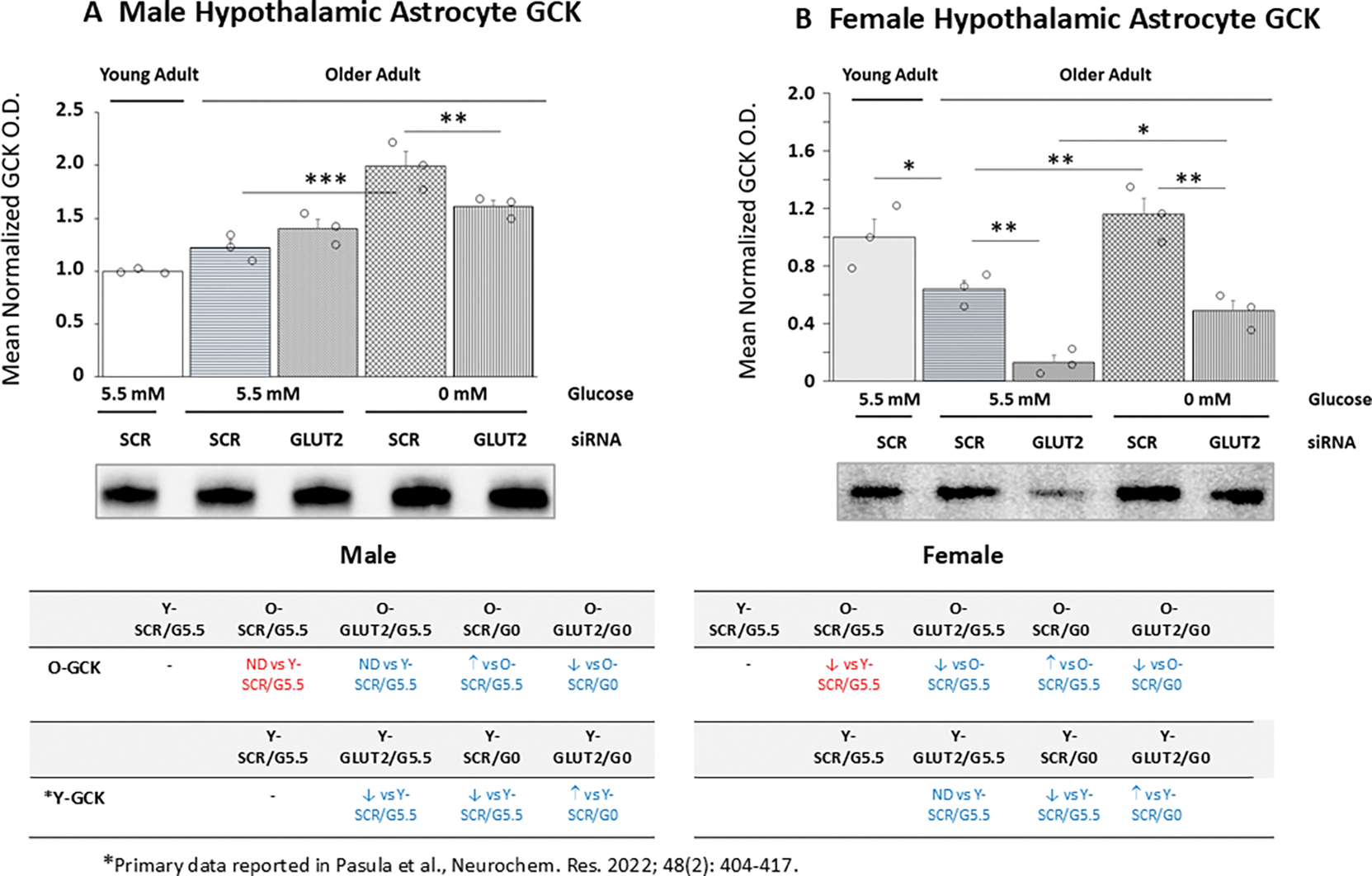
GLUT2 siRNA Effects on Glucokinase (GCK) Protein Expression in Old Glucose-Supplied or -Deprived Male versus Female Hypothalamic Primary Astrocytes. Old SCR or GLUT2 siRNA-pretreated male and female rat astrocyte cultures were incubated in the presence (G5.5) or absence (G0) of glucose; young adult male or female SCR siRNA/G5.5 astrocyte cultures served as controls. Data show mean normalized GCK protein O.D. ± S.E.M. for male (Figure 2A) and female (Figure 2B) astrocyte treatment groups. Statistical differences between discrete pairs of treatment groups are denoted as follows: *p < 0.05; **p < 0.01; ***p < 0.001. Tables at the bottom of Figure 3 summarize treatment effects on old male (at left) or female (at right) astrocyte culture GCK protein expression; results from earlier work involving SCR siRNA/G5.5, GLUT2 siRNA/G5.5, SCR siRNA/G0, or GLUT2 siRNA/G0 treatment effects on young adult astrocyte protein profiles are shown for comparison. Red font denotes a sex difference in age effect on SCR siRNA/G5.5 control group GCK protein expression. Blue font indicates an age-related change in treatment effect on GCK protein profiles.

Data in Figure 2 show effects of GLUT2 siRNA treatment alone or prior to glucose withdrawal on old male (Figure 2A) and female (Figure 2B) hypothalamic primary astrocyte GCK protein expression. Comparison of basal GCK protein profiles between young and old SCR siRNA/G5.5 cultures shows that this protein profile is comparable in young and old male astrocytes [*p*= 0.065], but that expression was significantly lower in older female young female cultures [*p*< 0.05]. Data show that GLUT2 gene knockdown did not affect old male astrocyte GCK protein expression (Figure 2A) but decreased this protein profile in old female rats (Figure 2B). Incubation in media lacking glucose caused up-regulated GCK protein levels in old astrocyte cultures from each sex; this stimulatory response was averted by GLUT2 gene knockdown pretreatment. The table *at lower left* indicates that in the male, aging affected GCK protein responses to GLUT2 siRNA, glucoprivation alone, and combinatory GLUT2 siRNA/glucose withdrawal. The table *at lower right* shows that astrocyte cultures from old female rats exhibited similar differences in GCK protein expression under those conditions compared to young female animals.

**Figure 3.**
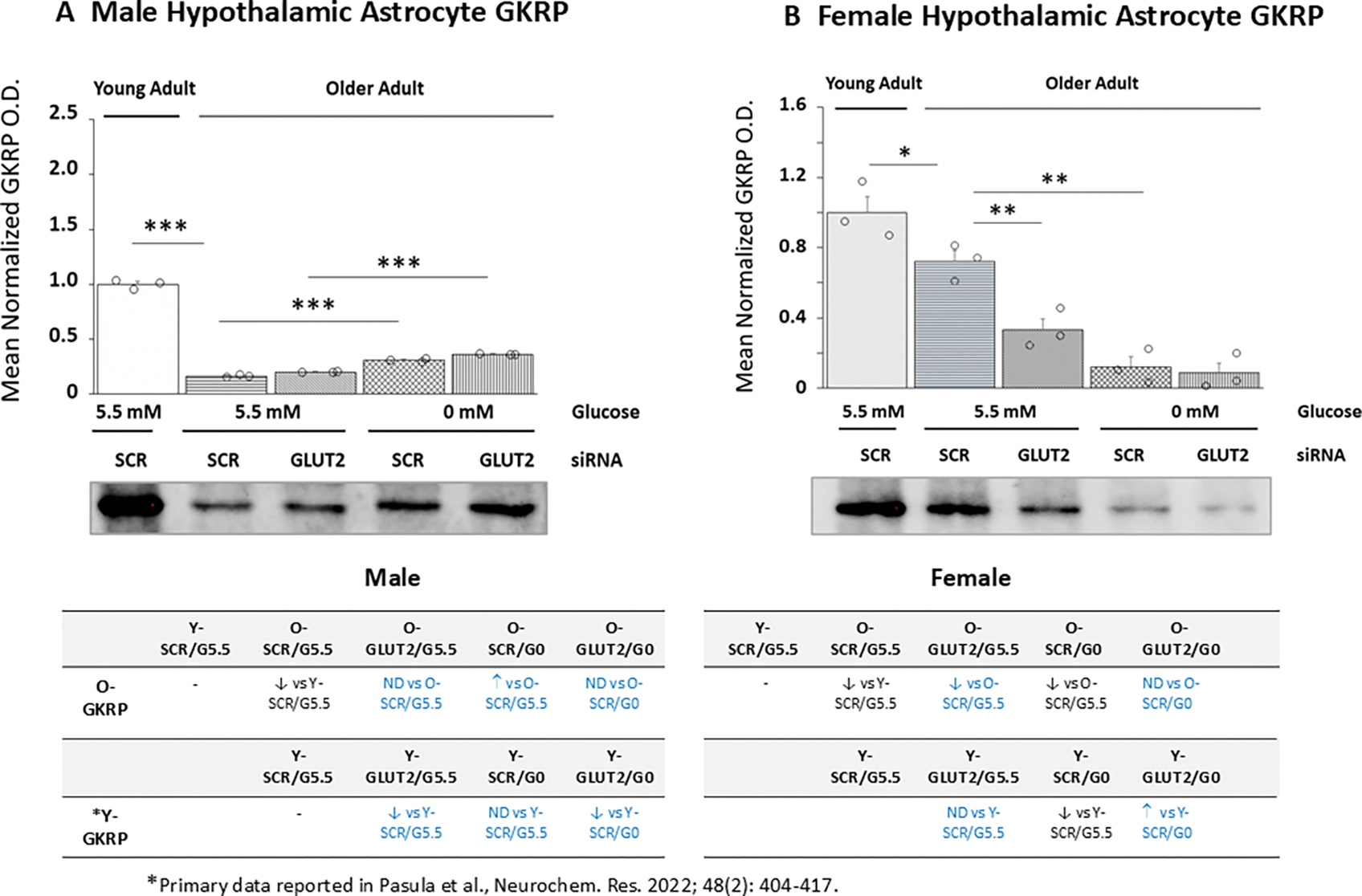
Glucokinase-Regulatory Protein (GKRP) Protein Expression in Old Male or Female Hypothalamic Primary Astrocytes: Effects of GLUT2 Gene Silencing. Old SCR or GLUT2 siRNA-pretreated male and female rat astrocyte cultures were incubated in the presence (G5.5) or absence (G0) of glucose; young adult male or female SCR siRNA/G5.5 astrocyte cultures served as controls. Normalized target protein measures are presented here as group mean values ± S.E.M. for male (Figure 3A) and female (Figure 3B) astrocyte treatment groups. Statistical differences between discrete pairs of treatment groups are denoted as follows: *p < 0.05; **p < 0.01; ***p < 0.001. Tables at the bottom of Figure 3 summarize treatments effects on old male (at left) or female (at right) astrocyte GKRP protein expression; results from earlier work involving SCR siRNA/G5.5, GLUT2 siRNA/G5.5, SCR siRNA/G0, or GLUT2 siRNA/G0 treatment effects on young adult astrocyte protein profiles are shown for comparison. Red font denotes a sex difference in age effect on SCR siRNA/G5.5 control group GKRP protein expression. Blue font indicates an age-related change in treatment effect on GKRP protein profiles.

Effects of GLUT2 gene silencing on GKRP protein levels in glucose-supplied or -deprived old male (Figure 3A) and female (Figure 3B) astrocytes are presented in Figure 3. Data indicate that baseline GKRP profiles were significantly different between young versus old animals of each sex [male: *p*< 0.001; female: *p*<0.05]. GLUT2 gene knockdown did not affect basal GKRP levels in old male rats but caused significant reductions in this protein in old female rat cultures. Glucose deprivation caused opposite changes in GKRP protein levels in old male (up-regulate) versus female (down-regulated) astrocyte cultures. GLUT2 siRNA pretreatment did not affect glucoprivation-associated GKRP expression profiles in old male or female astrocytes. Outcomes illustrated in tables at *lower left* (male) and *lower right* (female) indicate that aged astrocytes of each sex show differences in GLUT2 regulatory effects on basal and glucoprivic GKRP expression patterns compared to young adult rat cultures. Moreover, old male astrocyte cultures exhibit a gain in GKRP protein responsiveness to glucose withdrawal.

**Figure 4.**
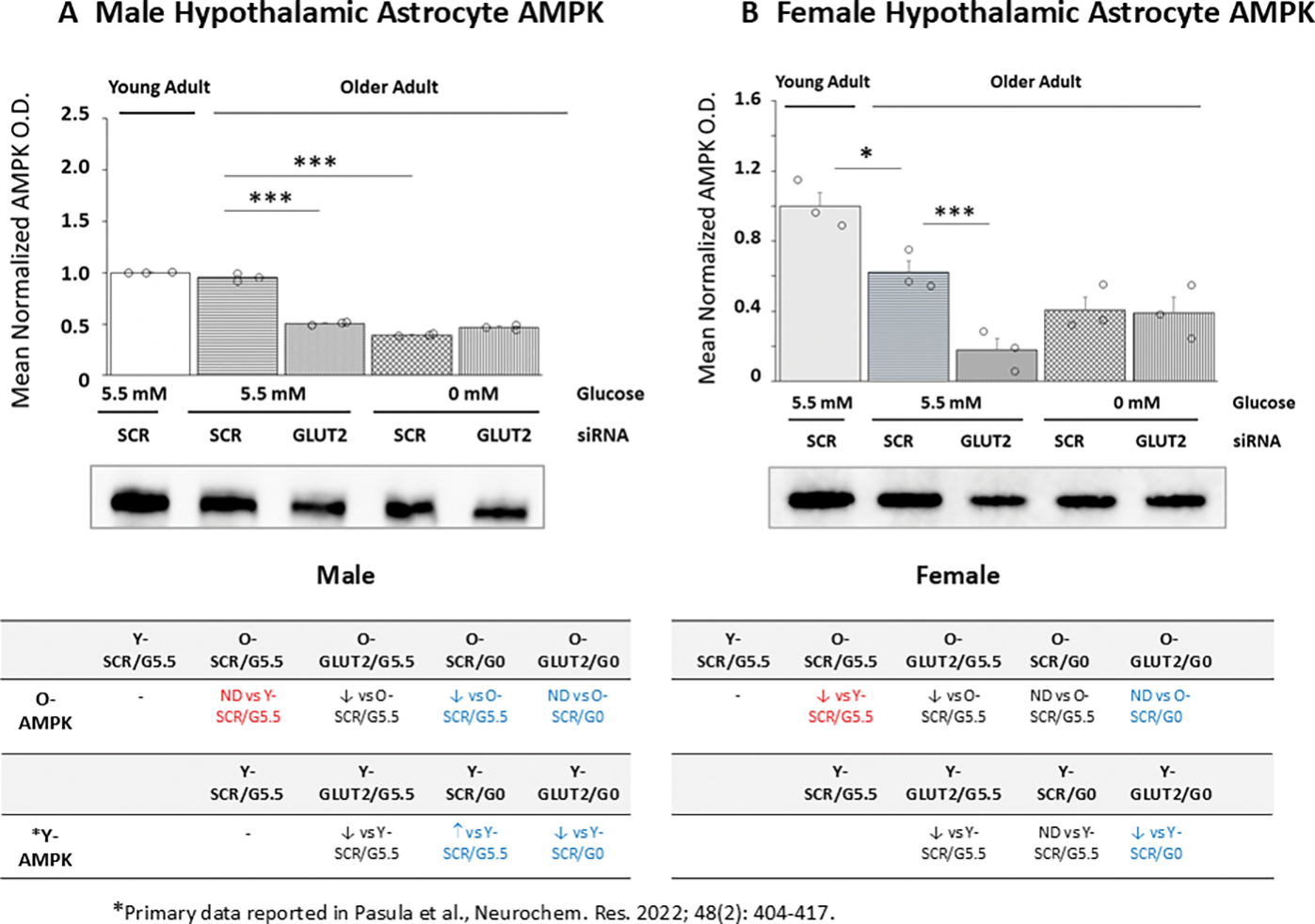
GLUT2 Gene Silencing Effects on 5’-AMP-Activated Protein Kinase (AMPK) Protein Expression in Old Male or Female Hypothalamic Primary Astrocytes. Old SCR or GLUT2 siRNA-pretreated astrocytes of each sex were incubated in the presence (G5.5) or absence (G0) of glucose; young adult male or female SCR siRNA/G5.5 astrocyte cultures served as controls. Normalized target protein measures are depicted here as group mean values ± S.E.M. for male (Figure 4A) and female (Figure 4B) treatment groups. Statistical differences between discrete pairs of treatment groups are denoted as follows: *p < 0.05; **p < 0.01; ***p < 0.001. Tables at the bottom of Figure 4 summarize treatments effects on old male (at left) or female (at right) astrocyte AMPK protein expression; results from earlier work involving SCR siRNA/G5.5, GLUT2 siRNA/G5.5, SCR siRNA/G0, or GLUT2 siRNA/G0 treatment effects on young adult astrocyte protein profiles are shown for comparison. Red font denotes a sex difference in age effect on SCR siRNA/G5.5 control group AMPK protein expression. Blue font indicates an age-related change in treatment effect on AMPK protein profiles.

Figure 4 depicts effects of GLUT2 gene silencing on old male (Figure 4A) or female (Figure 4B) rat hypothalamic primary astrocyte AMPK protein expression. Young and old SCR siRNA/G5.5-treated astrocytes had equivalent AMPK protein content [*p*= 0.158], whereas aged female astrocyte cultures had lower baseline AMPK protein compared to young control cultures [*p*< 0.05]. GLUT2 siRNA caused significant down-regulation of this protein profile in old male (Figure 4A) and female astrocytes. GLUT2 siRNA significantly reduced baseline AMPK protein content in old male and female astrocytes. Incubation in glucose-free media resulted in down-regulated AMPK profiles in old male, but not female astrocyte cultures. GLUT2 gene knockdown did not affect AMPK expression in glucose-deprived old astrocytes cultures derived from either sex. Tables at *bottom left* (male) and *bottom right* (female) indicate that in each sex, aging causes a loss of GLUT2 control of AMPK protein expression during glucoprivation; moreover, old male rat cultures exhibit a directional opposite shift in AMPK protein response to glucose deprivation, i.e., from up- (young astrocytes) to down- (old astrocytes) regulation.

**Figure 5.**
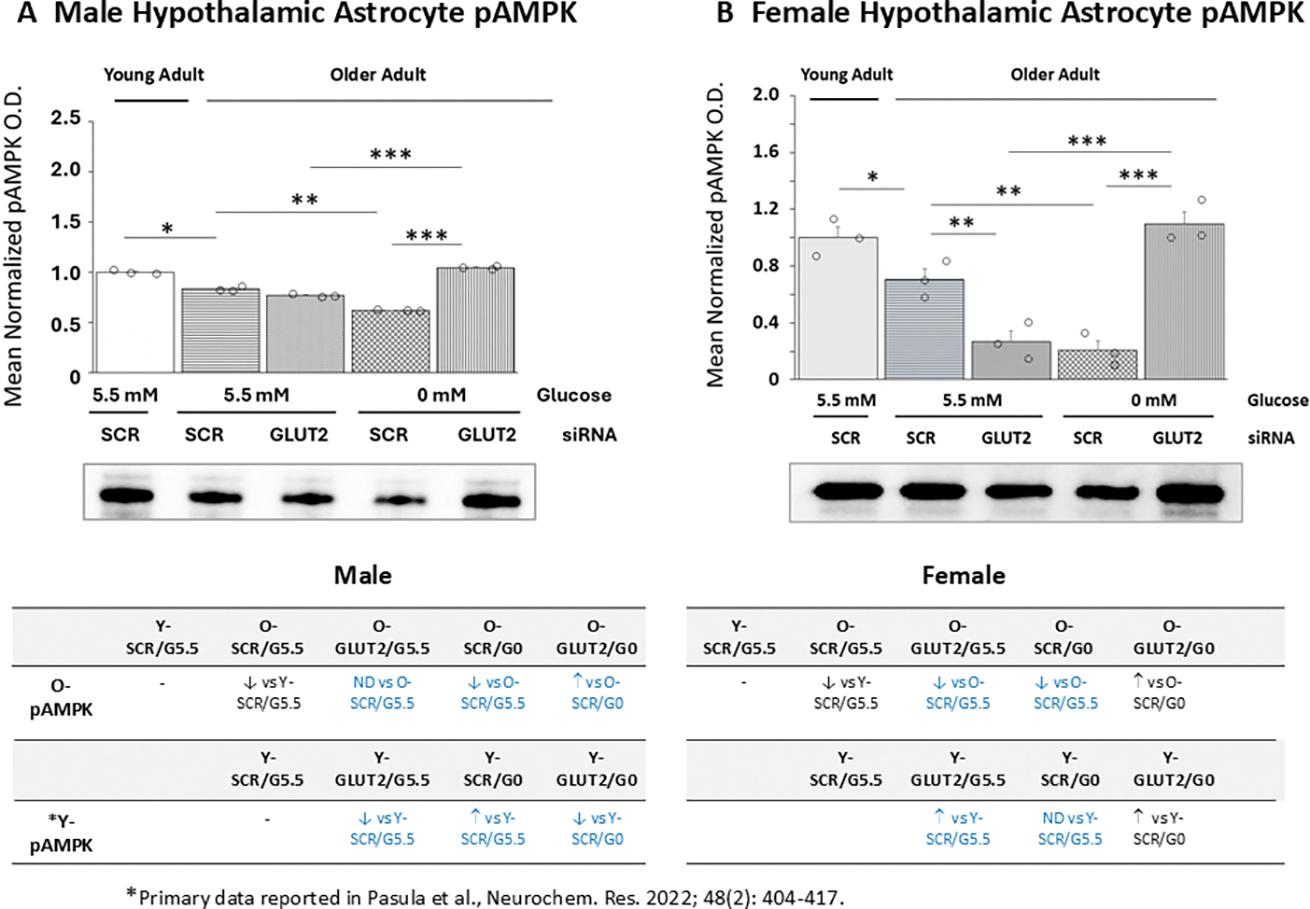
GLUT2 Gene Silencing Effects on phosphorylated AMPK (pAMPK) Protein Expression in Old Male or Female Hypothalamic Primary Astrocytes. Old SCR or GLUT2 siRNA-pretreated astrocytes of each sex were incubated in the presence (G5.5) or absence (G0) of glucose; young adult male or female SCR siRNA/G5.5 astrocyte cultures served as controls. Normalized target protein measures depicted here as group mean values ± S.E.M. for male (Figure 5A) and female (Figure 5B) treatment groups. Statistical differences between discrete pairs of treatment groups are denoted as follows: *p < 0.05; **p < 0.01; ***p < 0.001. Tables at the bottom of Figure 5 summarize treatments effects on old male (at left) or female (at right) astrocyte pAMPK protein expression; results from earlier work involving SCR siRNA/G5.5, GLUT2 siRNA/G5.5, SCR siRNA/G0, or GLUT2 siRNA/G0 treatment effects on young adult astrocyte protein profiles are shown for comparison. Red font indicates a sex difference in age effect on SCR siRNA/G5.5 control group pAMPK protein expression. Blue font denotes an age-related change in treatment effect on pAMPK protein profiles.

Data in Figure 5 illustrate GLUT2 gene silencing effects on pAMPK protein expression patterns in glucose-supplied versus -deprived old male (Figure 5A) and female (Figure 5B) hypothalamic primary astrocyte cultures. In each sex, basal pAMPK protein content was significantly decreased in old versus young astrocyte cultures [male: *p*< 0.05; female: *p*< 0.05]. Old male rat astrocyte cultures exhibited no change in pAMPK content after exposure to GLUT2 siRNA, yet female cultures showed down-regulated pAMPK expression. Glucose withdrawal caused significant down-regulation of pAMPK protein expression in old astrocytes of each sex; this inhibitory response was reversed by GLUT2 siRNA pretreatment in male and female cultures. The table at *bottom left*) shows that aging in the male shifts the direction of astrocyte pAMPK protein reactivity to glucoprivation from stimulatory to inhibitory and that this negative response is prevented by GLUT2 siRNA pretreatment. The table at *bottom right* indicates that GLUT2 regulation of baseline pAMPK protein differ between young (inhibitory) and old (stimulatory) female astrocyte cultures. Aging also causes a gain in female astrocyte pAMPK reactivity to glucoprivation.

**Figure 6.**
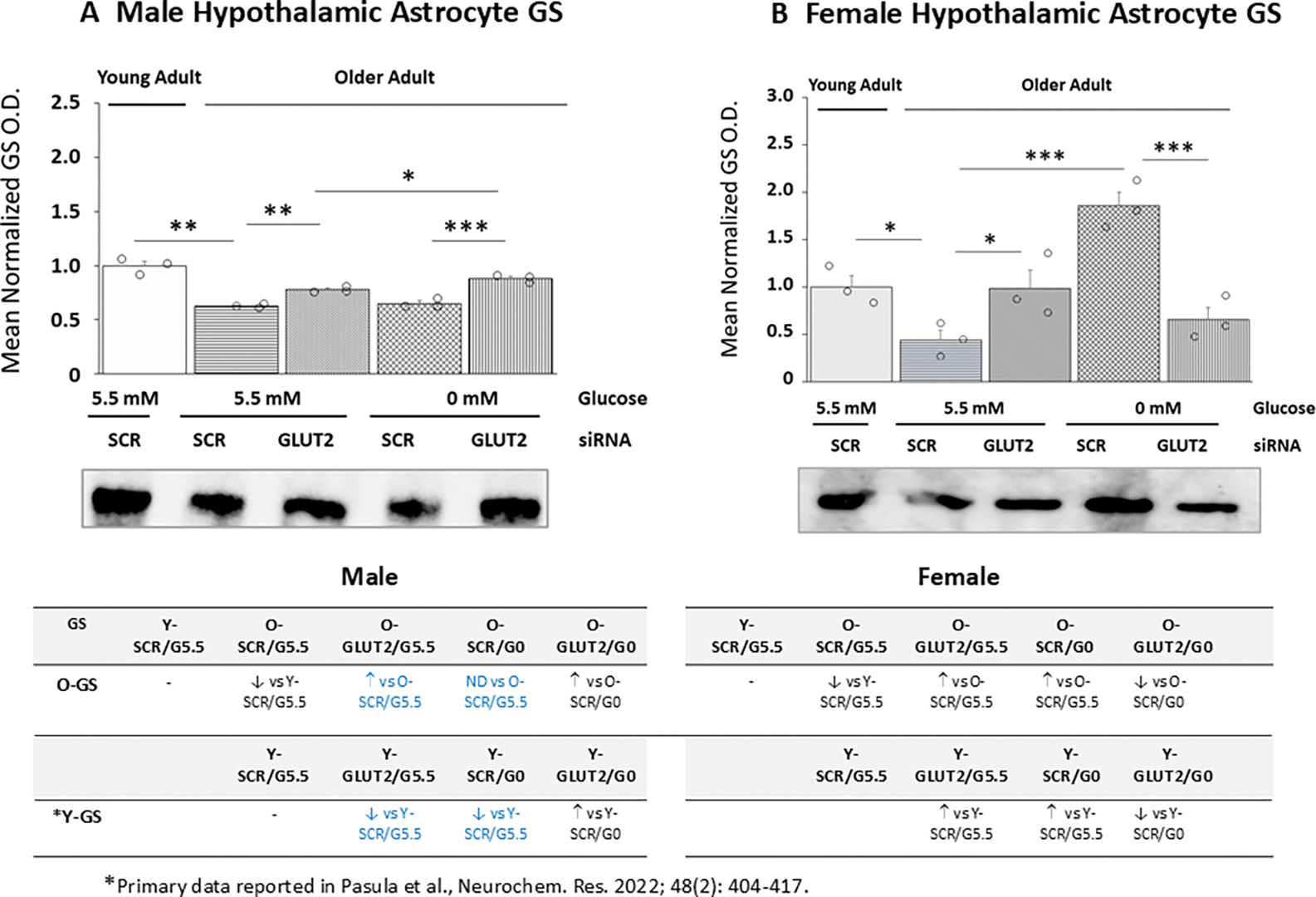
GLUT2 Gene Silencing Effects on Glycogen Synthase (GS) Protein Profiles in Old Male or Female Hypothalamic Primary Astrocytes. Old SCR or GLUT2 siRNA-pretreated astrocytes of each sex were incubated in the presence (G5.5) or absence (G0) of glucose; young adult male or female SCR siRNA/G5.5 astrocyte cultures served as controls. Normalized target protein measures are depicted here as group mean values ± S.E.M. for male (Figure 6A) and female (Figure 6B) treatment groups. Statistical differences between discrete pairs of treatment groups are denoted as follows: *p < 0.05; **p < 0.01; ***p < 0.001. Tables at the bottom of Figure 6 summarize treatments effects on old male (at left) or female (at right) astrocyte GS protein expression; results from earlier work involving SCR siRNA/G5.5, GLUT2 siRNA/G5.5, SCR siRNA/G0, or GLUT2 siRNA/G0 treatment effects on young adult astrocyte protein profiles are shown for comparison. Red font indicates a sex difference in age effect on SCR siRNA/G5.5 control group GS protein expression. Blue font denotes an age-related change in treatment effect on GS protein profiles.

Effects of GLUT2 gene knockdown on old male (Figure 6A) and female (Figure 6B) primary astrocyte GS protein expression are shown in Figure 6. Data indicate that in each sex baseline GS protein levels were significantly lower in old versus young SCR siRNA/G5.5 control cultures [male: *p*< 0.01; female: *p*< 0.05]. In old male (Figure 6A) and female (Figure 6B) glucose-supplied astrocyte cultures, GLUT2 siRNA increased GS protein expression. Glucoprivation did not affect old male astrocyte GS content but increased this protein profile in old female cultures. GLUT2 siRNA pretreatment had divergent effects on old glucose-deprived male (increased) versus female (decreased) astrocytes. The table at *bottom left* reveals age-related changes in GLUT2 and glucoprivic regulation of basal GS protein expression in old male astrocytes.

**Figure 7.**
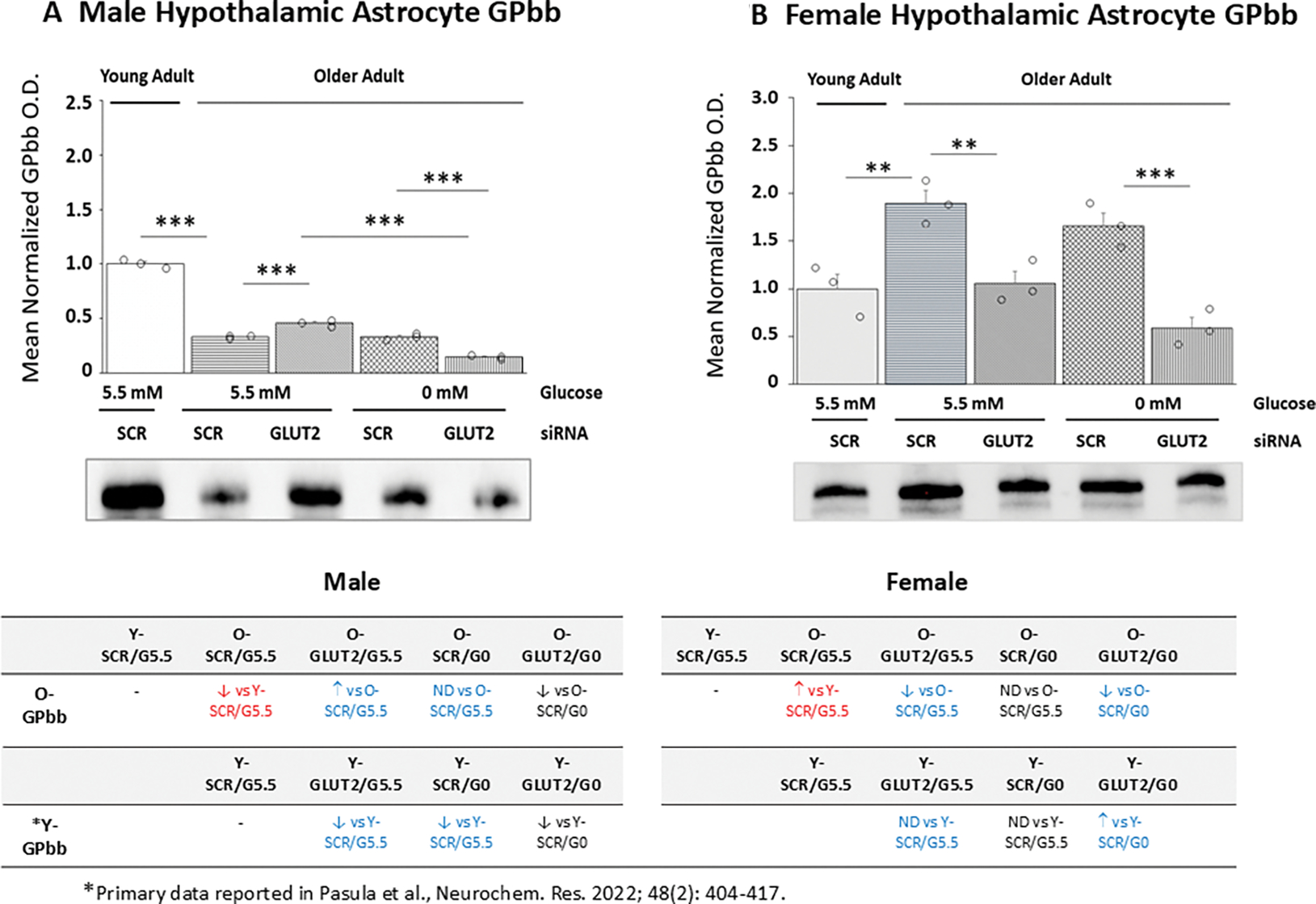
Glycogen Phosphorylase-Brain Type (GPbb) Protein Expression in Old SCR or GLUT2 siRNA-Pretreated Glucose-Supplied or -Deprived Hypothalamic Astrocytes. Old SCR or GLUT2 siRNA-pretreated astrocytes of each sex were incubated in the presence (G5.5) or absence (G0) of glucose; young adult male or female SCR siRNA/G5.5 astrocyte cultures served as controls. Normalized target protein measures are presented here as group mean values ± S.E.M. for male (Figure 7A) and female (Figure 7B) treatment groups. Statistical differences between discrete pairs of treatment groups are denoted as follows: *p < 0.05; **p < 0.01; ***p < 0.001. Tables at the bottom of Figure 7 summarize treatments effects on old male (at left) or female (at right) astrocyte GPbb protein expression; results from earlier work involving SCR siRNA/G5.5, GLUT2 siRNA/G5.5, SCR siRNA/G0, or GLUT2 siRNA/G0 treatment effects on young adult astrocyte protein profiles are shown for comparison. Red font indicates a sex difference in age effect on SCR siRNA/G5.5 control group GPbb protein expression. Blue font denotes an age-related change in treatment effect on GPbb protein profiles.

Figure 7 depicts effects of GLUT2 gene knockdown on GPbb protein expression patterns in old male (Figure 7A) and female (Figure 7B) primary hypothalamic astrocyte cultures. Data indicate that baseline astrocyte GPbb protein levels were significantly decreased (male; *p*< 0.001) or increased (female; *p*< 0.01) because of aging. GLUT2 gene silencing had sex-specific effects on astrocyte GPbb protein expression, i.e., causing up- or down-regulation in male versus female respectively. GLUT2 siRNA treatment in the presence of glucose elevated GPbb protein in old male, but not female cultures. Glucoprivation had no impact on this protein profile in old astrocytes of ether sex; nonetheless, GLUT2 gene knockdown caused a reduction in glucoprivic patterns of GPbb protein expression in old male and female astrocytes. Tables at *bottom left* (male) and *bottom right* (female) illustrate age-associated sex-dependent adjustments astrocyte GPbb profiles.

**Figure 8.**
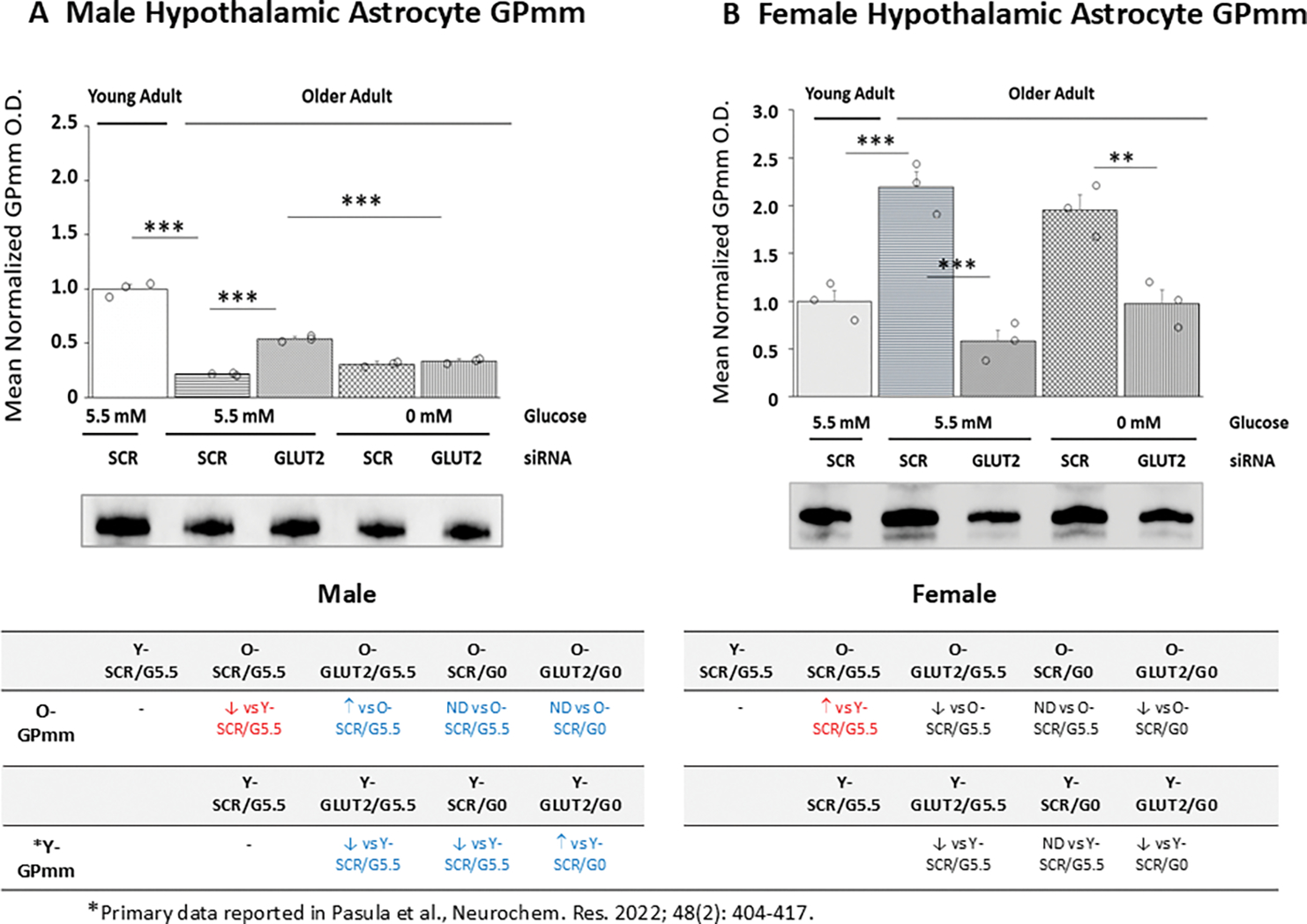
GLUT2 Gene Knockdown Effects on Old Male or Female Rat Hypothalamic Astrocyte Glycogen Phosphorylase-Muscle Type (GPmm) Protein Expression. Old SCR or GLUT2 siRNA-pretreated astrocytes of each sex were incubated in the presence (G5.5) or absence (G0) of glucose;. young adult male or female SCR siRNA/G5.5 astrocyte cultures served as controls. Normalized target protein measures are presented here as group mean values ± S.E.M. for male (Figure 7A) and female (Figure 7B) treatment groups. Statistical differences between discrete pairs of treatment groups are denoted as follows: *p < 0.05; **p < 0.01; ***p < 0.001. Tables at the bottom of Figure 8 summarize treatments effects on old male (at left) or female (at right) astrocyte GPmm protein expression; results from earlier work involving SCR siRNA/G5.5, GLUT2 siRNA/G5.5, SCR siRNA/G0, or GLUT2 siRNA/G0 treatment effects on young adult astrocyte protein profiles are shown for comparison. Red font indicates a sex difference in age effect on SCR siRNA/G5.5 control group GPmm protein expression. Blue font denotes an age-related change in treatment effect on GPmm protein profiles.

Effects of GLUT2 gene silencing on glucoprivic patterns of old male (Figure 8A) and female (Figure 8B) hypothalamic astrocyte GPmm protein expression are depicted in Figure 8. Old male and female cultures exhibited lower (male; *p<*0.001) or higher (female; *p<*0.001) GPmm protein content compared to young SCR siRNA/G5.5 control astrocytes. GLUT2 siRNA elevated (male) or reduced (female) GPmm protein expression in glucose-supplied astrocytes. Glucose starvation did not affect this protein profile in old astrocytes of either sex; glucoprivic patterns of GPmm protein expression in old female cultures were down-regulated by prior exposure to GLUT2 siRNA. The tables at *bottom left* and *right* show that old male astrocytes exhibit changes in GLUT2 and glucoprivic regulation of GPmm protein expression, whereas aging did not alter female astrocyte GPmm protein responses to those stimuli.

**Figure 9.**
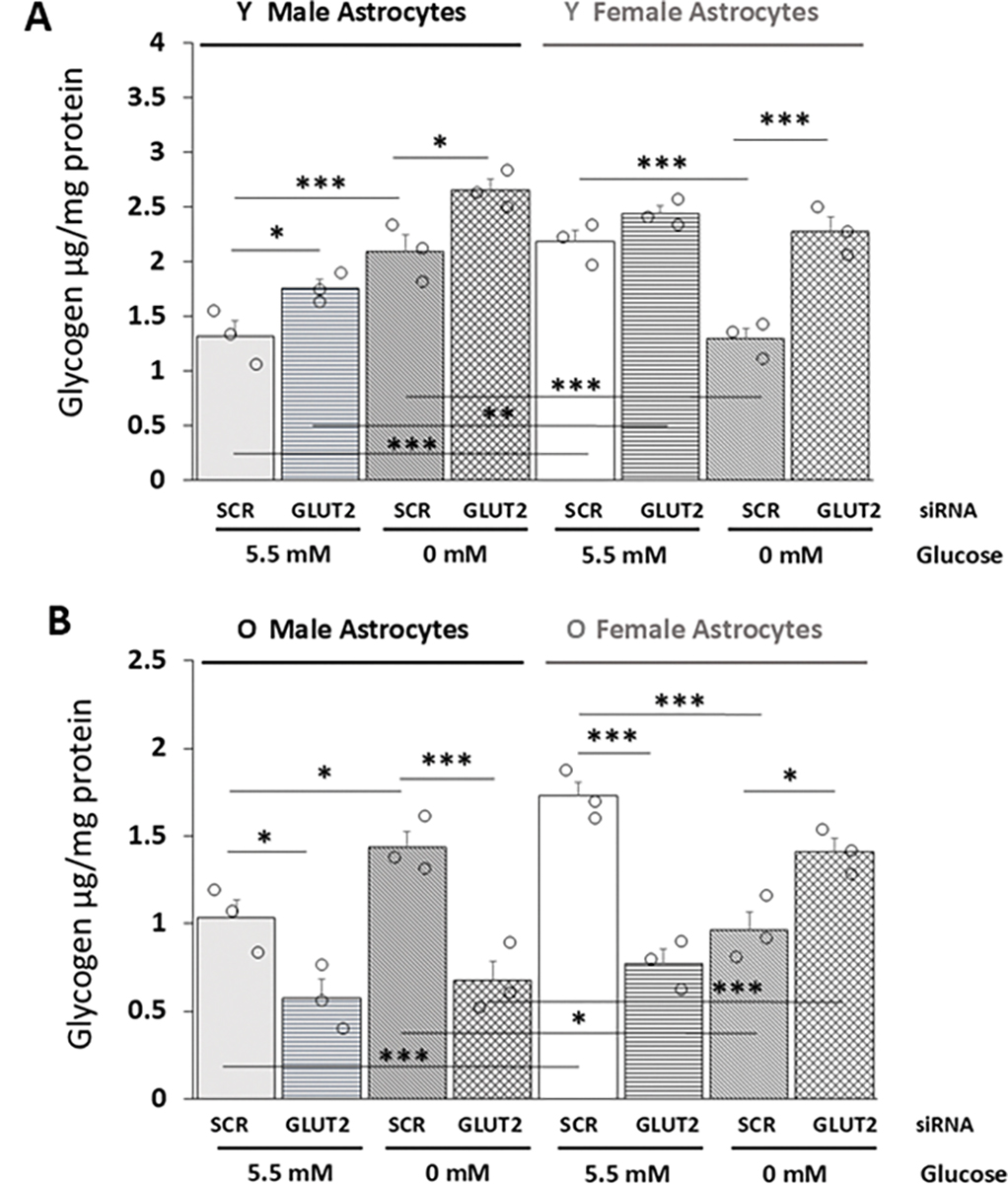
GLUT2 Gene Knockdown Effects on Young versus Old Hypothalamic Astrocyte Glycogen Concentrations. Data in Figures 9A and 9B depict mean glycogen concentrations for corresponding groups of young or old SCR or GLUT2 siRNA-pretreated astrocytes incubated in the presence (G5.5) or absence (G0) of glucose. In each figures, bars 1–4 *at left* and bars 5–8 *at right* depict data for male and female astrocytes organized into the following treatment groups: SCR siRNA/G5.5 [gray (male) or white (female) solid bars], GLUT2 siRNA/G5.5 [gray (male) or white (female) horizontal-striped bars], SCR siRNA/G0 [gray (male) or white (female) diagonal-striped bars], GLUT2 siRNA/G0 [gray (male) or white (female) crosshatched bars]. Astrocyte glycogen content was measured by LC-ESI-MS and expressed relative to cell protein and analyzed among treatment groups of each age by three-way ANOVA and Student-Newman-Keuls *post-hoc* test. Statistical differences between treatment group pairs by the following symbols: *p < 0.05; **p < 0.01; ***p < 0.001.

Figure 9 depicts outcomes of LC-ESI-MS analysis of young (Figure 9A; male treatment groups: bars 1–4 *at right*, female treatment groups: bars 5–8 *at left*) versus old (Figure 9B; male treatment groups: bars 1–4 *at left*, female treatment groups: bars 5–8 *at right*) rat astrocyte glycogen concentrations. Data in Figure 9A show that GLUT2 gene silencing up-regulated glycogen accumulation in glucose-deprived young male astrocytes (GLUT2 siRNA/G5.5 versus SCR siRNA G5.5), whereas young female astrocytes were resistant to this treatment. Glucoprivation had opposite effects on young male (increased) versus female (decreased) astrocyte glycogen content; GLUT2 siRNA pretreatment elevated glycogen levels in glucose-deprived cultures of either sex.

Data in Figure 9B show that glucose-supplied old astrocytes of each sex exhibited down-regulated glycogen concentrations in response to GLUT2 gene silencing. Incubation in glucose-free media caused augmentation (male) or diminution (female) of old astrocyte glycogen levels. These divergent sex-specific responses were each reversed by GLUT2 siRNA pretreatment. Outcomes disclose an aging-related shift in GLUT2 regulation of glycogen accumulation in male hypothalamic astrocytes, i.e., inhibitory (young) to stimulatory (old), and a gain in GLUT2 control of glycogen levels in glucose-supplied old female cultures.

## 4. Discussion

Glucogenic energy metabolism in the brain is compartmentalized by cell type, with astrocytes executing critical functions of glucose uptake, storage, and catabolism [10]. Astrocyte-neuron metabolic association impacts hypothalamic control of glucose counterregulation [14]. Aging results in counterregulatory dysfunction [49–54], but effects of this natural process on hypothalamic astrocyte glucose handling are unclear. The membrane glucose transporter-sensor GLUT2 exerts sex-contingent control of astrocyte metabolic sensing and glycogen metabolism in young adult rats [56]. Current research identifies distinctive sex-monomorphic versus -dimorphic changes in baseline and glucoprivic patterns of hypothalamic astrocyte glucose and ATP sensor marker protein and glycogen metabolic enzyme protein expression profiles and denotes if and how GLUT2 regulation of these proteins may change with age in each sex. Study outcomes infer that aging may affect critical hypothalamic astrocyte metabolic sensory cues and glycogen energy reserve volume and mobilization, according to sex, which may, in turn, influence astrocyte operations that are critical to neuro-metabolic stability.

Age-associated reductions in female astrocyte culture GLUT2 protein expression presumably correlate with decreased glucose uptake by that specific transporter. Present studies do not shed light on whether concurrent compensatory adjustments in expression of other GLUT family proteins occur, thus stabilizing net glucose internalization in this sex. An intriguing prospect is that age-associated reductions in GLUT2 protein expression may reflect, in part, a shift toward greater reliance on non-glucogenic versus glucogenic metabolic substrates for energy production in this sex. This notion is supported by current evidence that baseline expression of the initial and rate-limiting glycolytic pathway enzyme GCK is coincidently down-regulated in old female astrocytes. Interestingly, old male astrocytes exhibit decreased GLUT2 protein expression during glucoprivation, which represents an age-associated reversal of response direction. Recent studies document the capability of hypothalamic astrocytes to generate endogenous glucose *in vivo* via dephosphorylation of glucose-6-phosphate [59]. Regarding the *in vitro* model used here, it remains to be determined if the paradoxical increase in GLUT2 protein expression observed in glucose-deprived young male astrocytes [56] reflects, in part, increased cellular endogenous glucose production and release in the absence of media glucose. Old male and female astrocytes both exhibit up-regulated GCK protein expression during glucoprivation. The prospect of elevated intracellular glucose monitoring and heightened glycolysis in the absence of media glucose infers that these cells may react to this metabolic challenge by increased conversion of non-glucogenic substrates to glucose for partitioning to energy metabolic pathways. Interestingly, present data show that GLUT2 function is critical for glucoprivic up-regulation of GCK profiles in each sex; this stimulatory effect is opposite to GLUT2-mediated inhibition of this protein in glucose-deprived young astrocytes. Old astrocyte GKRP protein profiles are respectively increased (male) or decreased (female) during glucoprivation by GLUT2-independent mechanisms. These responses represent an age-related gain in GKRP response to glucose deficiency (males) alongside loss of GLUT2 control of this protein (both sexes) under those conditions. GKRP forms complexes with glucose-free GCK to isolate non-functional enzyme inside the nucleus. Evidence here for up- or down-regulated expression of this protein in glucose-deprived old male or female astrocytes, respectively, may be indicative of divergent changes in proportional GCK occupancy by glucose. Lack of GLUT2 control of glucoprivic patterns of GKRP expression in old astrocytes implies that control of GCK molecules available for glucose binding may be governed by intracellular rather than plasma membrane glucose content.

Current results document age-associated adjustments in basal AMPK protein expression in old female, but not male hypothalamic astrocytes, yet reveal coincident down-regulation of pAMPK profiles in each sex. The prospect that the mean baseline pAMPK/AMPK expression ratio may be lowered by age in the male points to potential enhancement of net cellular energy stability. This presumption will require analytical verification that quantifiable ATP levels are increased in old versus young astrocyte SCR siRNA/G5.5 treatment groups. Old glucose-deprived male astrocytes exhibit parallel down-regulation of AMPK and pAMPK protein profiles, which suggests that net enzyme activity state may be unaffected by this metabolic stress. On the other hand, glucoprivation did not alter old female astrocyte AMPK expression but decreased pAMPK protein content, inferring that cellular energy balance may be relatively more positive compared to old female SCR siRNA/G5.5 controls. Current results also document aging-related loss of GLUT2 control of glucoprivic patterns of AMPK protein expression in each sex, as well as a stimulatory to inhibitory shift in GLUT2 regulation of pAMPK expression in male astrocytes.

Data illustrate age-associated down-regulated baseline expression of multiple target proteins, including GS, in female hypothalamic astrocytes, yet show elevated basal GPbb and GPmm protein expression in these cells. On the other hand, each glycogen metabolic enzyme protein profile was relatively lower in old versus young male astrocyte cultures. Glucoprivation enhanced GS expression in old female, but not male astrocytes, representing a loss of response in the latter sex. GLUT2 evidently exerts divergent control of this protein profile in glucose-deprived male (inhibitory) and female (stimulatory) astrocytes regardless of age. Inverse adjustments in baseline GPbb protein expression in old male or female astrocytes versus young SCR siRNA/G5.5 controls correlate with GLUT2 inhibition or stimulation, respectively; GLUT2 control of basal GPbb levels is thus altered with age. Glucose starvation did not alter GPbb protein expression in old male or female rat astrocytes, indicating a shift from GPbb inhibition in young male cultures. Aging evidently alters GLUT2 control of baseline (both sexes) and glucoprivic-associated (female only) GPbb protein levels. Old astrocytes likewise exhibit GLUT2 suppression (male) or augmentation (female) of basal astrocyte GPmm protein expression, and lack of GPmm protein reactivity to glucose withdrawal. Regulatory effects of GLUT2 or glucoprivation alone or in combination on old male astrocyte GPmm profiles are subject to aging-associated changes.

Aging has a unique, substantial impact on GLUT2 regulation of hypothalamic astrocyte glycogen concentrations in each sex but does not affect divergent effects of glucoprivation on glycogen accumulation in the two sexes. In young male astrocytes, this glucose transporter-sensor opposes glycogen expansion irrespective of the presence or absence of glucose, yet old male glucose-supplied or -deprived cultures exhibit GLUT2 augmentation of glycogen mass. These data depict a reversal of direction of control due to aging, implicating GLUT2 as a primary driver of glycogen expansion in glucoprivic male astrocytes instead limiting accumulation in young cultures. Thus, this sensor may exert negative (young) or positive (old) influence on the ratio of glycogen synthesis versus disassembly in male astrocytes, respectively, thereby curbing or promoting glucose storage. Basal glycogen amassment in old female astrocytes is likewise up-regulated by GLUT2, in contrast to a lack of this regulatory control in young female glia. During glucose sufficiency, old male and female astrocytes may release glucosyl units from the glycogen reserve for glycolytic processing to yield lactate in response to GLUT2 directive.

Glucoprivic amplification of old male astrocyte glycogen content occurs despite GS, GPbb, and GPmm protein insensitivity to this metabolic challenge. This discordance infers that glucose deficiency may exert differential control of total glycogen metabolic enzyme protein production versus enzyme activity of one of more of those proteins. Current work does reveal if specific of either enzyme is impacted by this metabolic stress. GS is active in the non-phosphorylated state owing to glucose 6-phosphate allosteric effects, yet GP enzyme isoforms are activated via phosphorylation or AMP allosteric action; it unclear if glucoprivation controls these post-transcriptional modifications in either sex. There is an urgent need to distinguish effects of GLUT2 or glucoprivic control alone or in combination GS, GPmm, and GPbb activation state relative to total protein expression as results could yield insights on how these distinctive stimuli may regulate glycogen turnover separate from mass during glucose sufficiency versus deficiency. It is unclear if astrocyte glycogen is a common substrate for GPbb- versus GPmm-mediated breakdown, or if it is spatially organized into distinct segments from which glucosyl units are liberated primarily by one GP variant. Further studies are needed to determine if GLUT2 causes comparable or dissimilar changes in GPbb compared to GPmm enzyme activity, and to examine how specific activity adjustments for individual GP isoforms may influence total glycogen mass. It is important to remember that efforts to resolve this critical issue are currently thwarted by non-availability of antibody-based analytical tools for quantification of GP variant phosphorylation.

A point that merits consideration is that the current *in vitro* metabolic fuel deficiency paradigm is not an exact replication of pathophysiological exogenous insulin-induced reductions in brain tissue glucose levels *in vivo* that may result from iatrogenic hypoglycemia. Another drawback of the experimental approach used here is the lack of inclusion of step-wise reductions in media glucose. Future research seeks to establish whether old male and female GLUT2 exhibits capabilities to detect and act upon small, physiological-like decreases or increases in glucose availability and, additionally, implement that sensory information to impose graded regulatory effects on expression of target protein evaluated here, including those that are refractory here to GLUT2 control in the absence of glucose. Recent *in vivo* studies show that hypothalamic GLUT2 regulates hypoglycemic patterns of hypothalamic astrocyte glucose and glycogen metabolic enzyme protein expression and systemic counterregulatory hormone secretion [63]. These findings bolster the need to expand present efforts by characterization of potential aging effects on *in vivo* hypothalamic astrocyte glucose and glycogen handling and consequential impacts on astrocyte-nerve cell metabolic coupling in each sex.

Another drawback of the present project involves use of whole-hypothalamic tissue as a source of astrocytes for primary culture, as averaged measures of glial cell function across this broad region may likely mask unique responses of local astrocyte populations to adjustments in glucose provision. *In vivo* studies allow for acquisition of brain cell samples at very high-neuroanatomical resolution for molecular analyses; a practice that is growing in recognition of structure-based diversity of function. Accordingly, there is a pressing need for development of methods for generation of astrocyte primary cultures from tissue comprising distinct hypothalamic structures of unique significance for neural regulation of glucostasis, i.e., ventromedial, dorsomedial, and arcuate nuclei and lateral hypothalamic area [1].

A fundamental unanswered question involves the molecular mechanisms whereby membrane GLUT2 may regulate hypothalamic astrocyte glycolytic pathway volume, glycogen turnover and mass, and cellular energy status. Current work raises the corollary issue of how aging may affect those processes. Recent studies identify sex-specific GLUT2 control of distinctive hypothalamic astrocyte PI3K/Akt/mTOR pathway protein expression and/or phosphorylation, and, moreover, characterize pathway components affected by glucose shortage and reveal whether those responses involve GLUT2-dependent and/or -independent mechanisms [60]. That work importantly implicates glucose status as an important determinant of direction, i.e., positive versus negative GLUT2 control of PI3K/Akt/mTOR pathway activity in each sex and GLUT2 as a driver of distinctive pathway protein reactions to glucoprivation in female, but not male. There is a valid necessity to determine if this signal transduction cascade executes documented GLUT control of astrocyte glucose and glycogen metabolism in young animals, and if so, how pathway operation may be affected by age in each sex. An additional consideration is whether GLUT2-controlled PI3K/Akt/mTOR regulatory actions may involve cross-talk mitogen-activated pathway kinase (MAPK) signaling. Our studies disclose that GLUT2 inhibits ERK1/2, p38, and SAPK/JNK MAPK activity in young adult male hypothalamic astrocytes but imposes distinctive stimulatory or inhibitory effects on these individual signaling pathways in female cultures [61]. Results also documented a stimulatory role for GLUT2 in p38 MAPK activation in glucose-starved female astrocytes but indicate that this sensor can act to suppress or trigger SAPK/JNK phosphorylation/activation in glucose-deprived male versus female glial cells, respectively [62].

Aging is associated with counterregulatory endocrine dysfunction and neurogenic unawareness of hypoglycemia, which exacerbates the risk of neurological impairment or injury [49–54]. Insight on how this pathophysiological mal-adaptation occurs is needed to appropriately focus efforts to develop pharmacotherapeutic approaches to mitigate such damage. Studies in young adults show that hypothalamic astrocyte-nerve cell metabolic coupling shapes neural control of counterregulation. Current evidence here for sex-dimorphic age-related changes in hypothalamic astrocyte glucose handling and glycogen amassment and mobilization should be extended by in vivo experimentation to determine, for each sex, if and how these critical astrocyte functions may be affected by age in a whole animal model and to characterize resultant impacts on counterregulation.

In summary, current studies used a hypothalamic primary astrocyte cell culture model in conjunction with Western blot and LC-MS techniques to address the notion that aging may cause sex-contingent changes in GLUT2-controlled metabolic monitoring and glycogen metabolic operations in this glial cell type. Research outcomes show GLUT2-dependent GCK augmentation in glucose-deprived astrocytes of each sex, unlike GLUT2 down-regulation of this protein in young astrocytes. Glucose starvation of old male astrocytes caused GLUT2-independent suppression of 5’-AMP-activated protein kinase (AMPK) protein, reflective of a loss of GLUT2 up-regulation of this protein with age. Glucoprivation caused GLUT2-mediated inhibition of phospho-AMPK profiles in each sex, differing from GLUT2-mediated glucoprivic augmentation of this protein in young male astrocytes and phospho-AMPK insensitivity to glucose starvation in young female cultures. GS and GP isoform proteins were refractory to glucoprivation in old male cultures, contrary to down-regulation of these proteins in young glucose-deprived male astrocytes. Aging elicited a shift from GLUT2 inhibition to stimulation of male astrocyte glycogen accumulation and caused gain of GLUT2 control of female astrocyte glycogen. Outcomes document sex-specific, aging-related alterations in GLUT2 control of hypothalamic astrocyte glucose and ATP monitoring and glycogen mass and metabolism. Results warrant future initiatives to assess how these adjustments in hypothalamic astrocyte function may affect neural operations that are shaped by astrocyte-neuron metabolic partnership.

## 5. Conclusion

Present outcomes emphasize the need for focus on potential aging effects on the functionality of astrocyte populations throughout the brain, including neural structures that rely upon glycogen mobilization to support critical neurological operations such as memory and learning. There is also a lack of insight on the onset, in males versus females, of adjustments in astrocyte glucose handling and glycogen metabolism in discrete loci in the hypothalamus and elsewhere, and on the molecular mechanisms that elicit such changes. Further research is needed to investigate the prospect that the magnitude of alterations in astrocyte-dependent neuro-metabolic stability may be incremental over all or a segment of the lifespan and may correlate, at a certain point, with impairments in neural regulation of somatic and autonomic functions, including maintenance of glucose homeostasis.

## Supplementary Material

1

## Data Availability

The data that support the findings of this study are available from the corresponding author upon reasonable request.

## Abbreviations:

AMPK: 5’ AMP-activated protein kinase*α*1/2.
CNS: central nervous system.
GCK: glucokinase.
GKRP: glucokinase regulatory protein.
GLUT2: glucose transporter-2.
GPbb: glycogen phosphorylase-brain type.
GPmm: glycogen phosphorylase-muscle type.
GS: glycogen synthase.
IIH: insulin-induced hypoglycemia.
LC-ESI-MS: uHPLC-electrospray ionization-mass spectrometry.
pAMPK: phosphoAMPK*α*1/2
