# Supplementary material for: Aging Effects on Metabolic Sensor and Glycogen Metabolism in Old Male versus Female Rat Primary Hypothalamic Astrocyte Cultures
Source: Neuroglia. Author manuscript; Available in PMC 2025 Dec 25. (PMC12732729; doi:10.3390/neuroglia6040041)
Supplement: 1 [file NIHMS2123422-supplement-1.pdf]

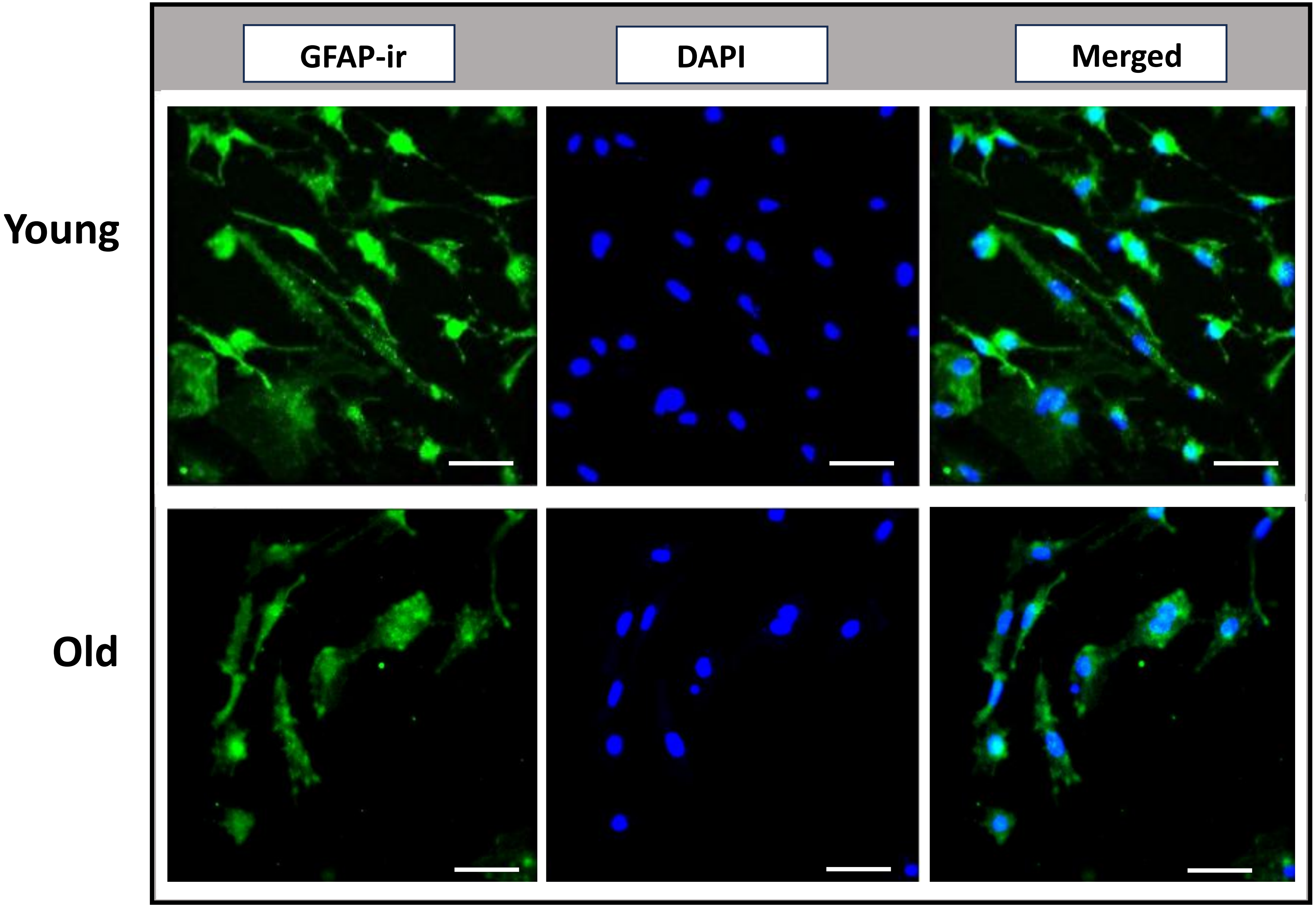

Supplementary Figure 1. Immunofluoresence Staining of Cultured Astrocytes for Astrocyte-Specific Marker Glial Fibrillary Acidic Protein (GFAP). Young (top row) and old (bottom row) hypothalamic astrocyte cultures were processed by immunocytochemistry (anti-GFAP; 1:1,000; Cell Signaling Technology, Danvers, MA; RRID:AB_561049) to detect cytoplasmic GFAP immunoreactivity (left-hand column; green fluorescence) and were stained with DAPI (middle column; blue fluorescence) label nuclear DNA. Images were obtained with Zeiss LSM 5 PASCAL confocal scanning laser microscope. Merged images are shown in the right-hand column. Scale bar = 50 microns.

Supplementary Figure 2

1A Uncropped Male Astrocyte GLUT2 Immunoblot

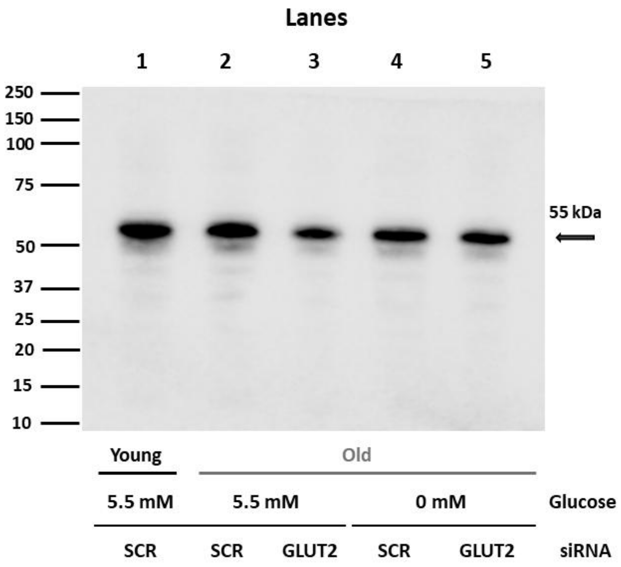

1B Uncropped Male Astrocyte GCK Immunoblot

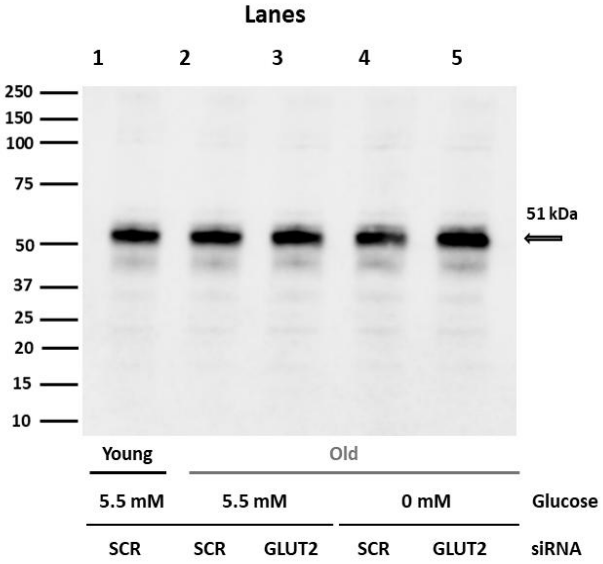

1C Uncropped Male Astrocyte GKRP Immunoblot

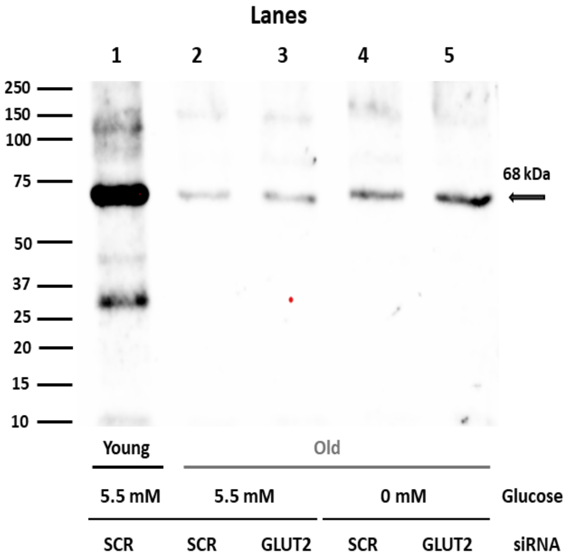

1D Uncropped Male Astrocyte AMPK Immunoblot

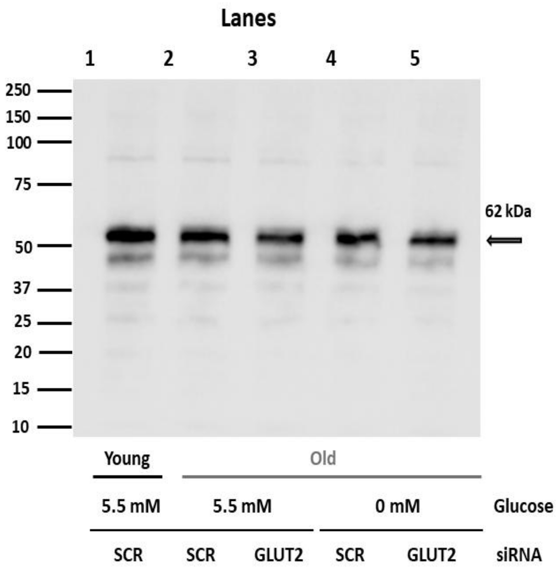

1E Uncropped Male Astrocyte pAMPK Immunoblot

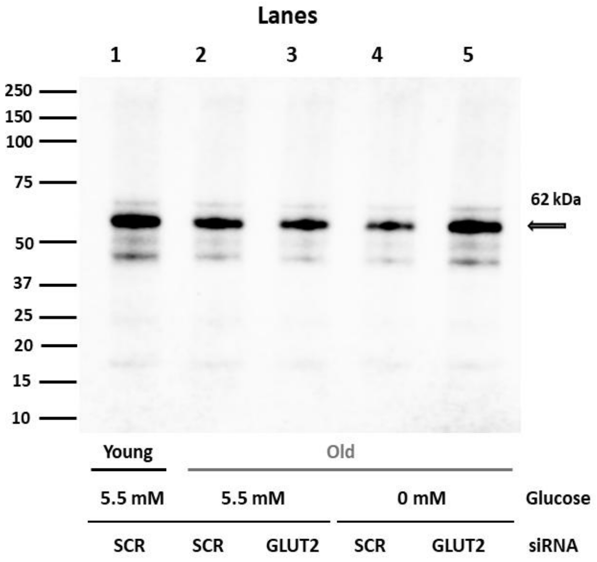

1F Uncropped Male Astrocyte GS Immunoblot

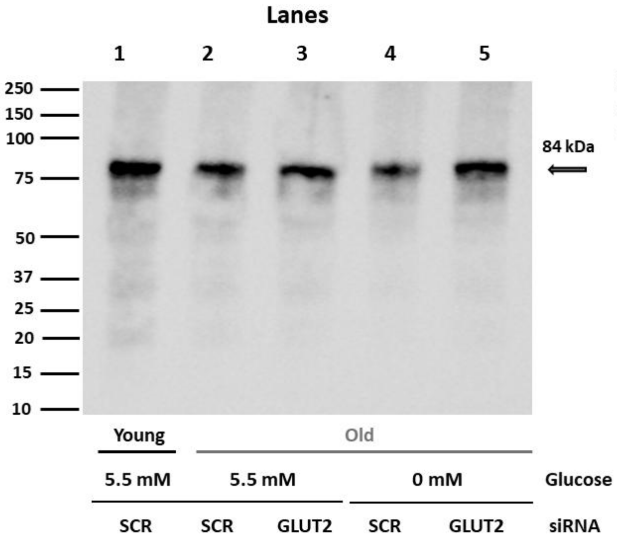

1G Uncropped Male Astrocyte GPbb Immunoblot

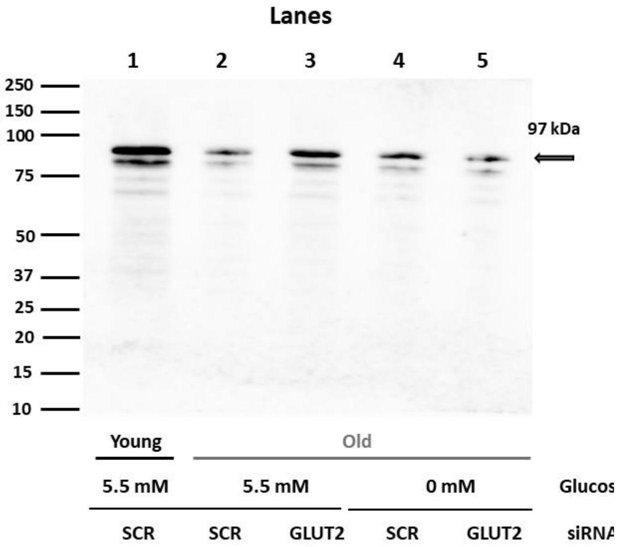

1H Uncropped Male Astrocyte GPmm Immunoblot

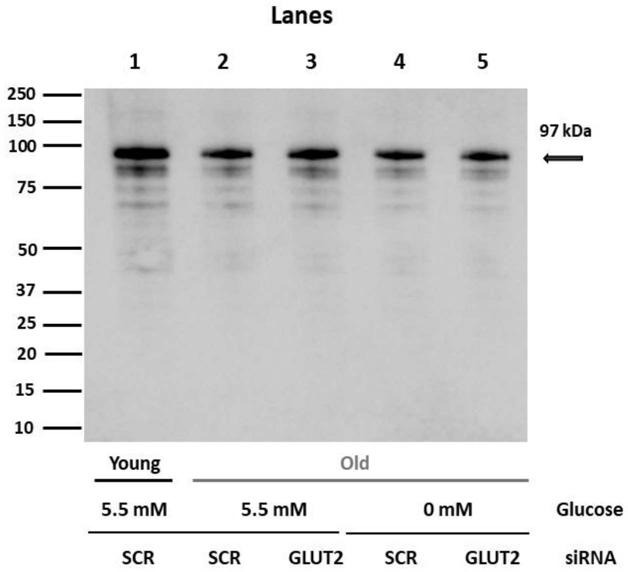

Legend to Supplementary Figure 2. Full-length, uncropped representative Western blots, corresponding to cropped images of immunodetectable young and old male rat hypothalamic primary astrocyte culture target proteins shown in Figures 1A, 2A, 3A, 4A, 5A, 6A, 7A and 8A. Old male primary hypothalamic astrocytes were pretreated with scramble (SCR) or GLUT2 siRNA prior to incubation in media supplemented with (5 mM) or lacking (0 mM) glucose. Control astrocyte cultures established from young adult male or female rats were exposed to SCR siRNA before incubation with 5.5 mM glucose-containing media.

Supplementary Figure 3

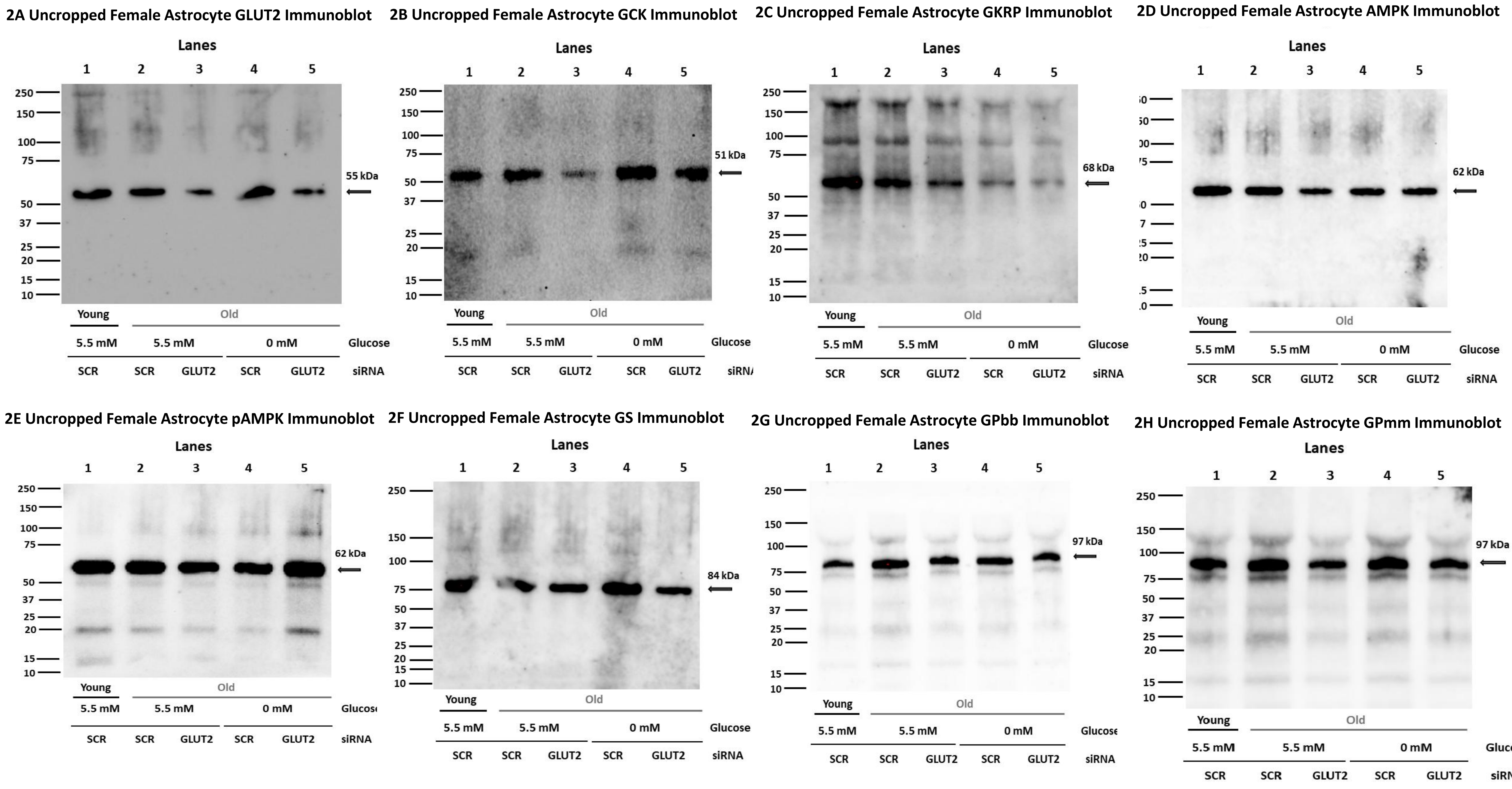

Legend to Supplementary Figure 3. Full-length, uncropped representative Western blots, corresponding to cropped images of immunodetectable young and old female rat hypothalamic primary astrocyte culture target proteins shown in Figures 1B, 2B, 3B, 4B, 5B, 6B, 7B, and 8B. Old male primary hypothalamic astrocytes were pretreated with scramble (SCR) or GLUT2 siRNA prior to incubation in media supplemented with (5 mM) or lacking (0 mM) glucose. Control astrocyte cultures established from young adult male or female rats were exposed to SCR siRNA before incubation with 5.5 mM glucose-containing media.

Supplementary Figure 4

Figure 1. Male: $F_{(3,8)}$= 116.94, *p*<0.001; Pretreatment primary effect: $F_{(1,8)}$= 216.43, *p*<0.001; Treatment primary effect: $F_{(1,8)}$= 4.16, *p*= 0.066; Treatment/pretreatment/interaction: $F_{(1,8)}$= 60.19, *p*<0.001. Female: $F_{(3,8)}$= 18.80, *p*<0.001; Pretreatment primary effect: $F_{(1,8)}$= 53.40, *p*<0.001; Treatment primary effect: $F_{(1,8)}$= 2.02, *p*=0.193; Treatment/pretreatment interaction: $F_{(1,8)}$= 0.99, *p*= 0.0348.

Figure 2. Male: $F_{(3,8)}$= 21.98, *p*<0.001; Pretreatment chief effect: $F_{(1,8)}$= 0.29, *p*=0.604; Treatment chief effect: $F_{(1,8)}$= 40.42, *p*<0.001; Treatment/pretreatment interaction: $F_{(1,8)}$= 15.58, *p*=0.002]. Female: $F_{(3,8)}$= 30.20, *p*<0.001; Pretreatment chief effect: $F_{(1,8)}$= 57.51, *p*<0.001; Treatment chief effect: $F_{(1,8)}$= 31.96, *p*<0.001; Treatment/pretreatment interaction: $F_{(1,8)}$= 1.13, *p*=0.320].

Figure 3. Male: $F_{(3,8)}$= 1.21, *p*=0.350; Pretreatment principal effect: $F_{(1,8)}$= 0.96, *p*=0.349; Treatment principal effect: $F_{(1,8)}$= 0.11, *p*=0.743; Treatment/pretreatment interaction: $F_{(1,8)}$= 1.72, *p*=0.217]. Female: $F_{(3,8)}$= 24.27, *p*<0.001; Pretreatment principal effect: $F_{(1,8)}$= 12.87, *p*=0.007; Treatment principal effect: $F_{(1,8)}$= 51.05, *p*<0.001; Treatment/pretreatment interaction: $F_{(1,8)}$= 8.91, *p*=0.017],

Figure 4. Male: $F_{(3,8)}$= 442.248, *p*<0.001; Pretreatment primary effect: $F_{(1,8)}$= 178.58, *p*<0.001; Treatment primary effect: $F_{(1,8)}$= 448.88, *p*<0.001; Treatment/pretreatment interaction: $F_{(1,8)}$= 336.67, *p*<0.001]. Female: [$F_{(3,8)}$= 6.05, *p*=0.019; Pretreatment primary effect: $F_{(1,8)}$= 9.80, *p*=0.014; Treatment primary effect: $F_{(1,8)}$= 0.00, *p*=0.990; Treatment/pretreatment interaction: $F_{(1,8)}$= 8.34, *p*=0.020.

Figure 5. Male: $F_{(3,8)}$= 26.30, *p*<0.001; Pretreatment chief effect: $F_{(1,8)}$= 16.09, *p*=0.002; Treatment chief effect: $F_{(1,8)}$= 0.08, *p*=0.784; Treatment/pretreatment interaction: $F_{(1,8)}$= 69.74, *p*<0.001]. Female: $F_{(3,8)}$= 29.99, *p*<0.001; Pretreatment chief effect: $F_{(1,8)}$= 8.87, *p*=0.018; Treatment chief effect: $F_{(1,8)}$= 4.74, *p*=0.061; Treatment/pretreatment interaction: $F_{(1,8)}$= 76.36, *p*<0.001].

Figure 6. Male: $F_{(3,8)}$= 1.40, *p*=0.294; Pretreatment principal effect: $F_{(1,8)}$= 1.56, *p*= 0.237; Treatment principal effect: $F_{(1,8)}$= 0.12, *p*= 0.733; Treatment/pretreatment interaction: $F_{(1,8)}$= 2.99, *p*= 0.112. Female: $F_{(3,8)}$= 18.43, *p*< 0.001; Pretreatment principal effect: $F_{(1,8)}$= 5.13, *p*= 0.053; Treatment principal effect: $F_{(1,8)}$= 13.98, *p*= 0.006; Treatment/pretreatment interaction: $F_{(1,8)}$= 36.19, *p*< 0.001].

Figure 7. Male: $F_{(3,8)}$= 3.27, *p*=0.063; Pretreatment primary effect: $F_{(1,8)}$= 2.21, *p*=0.165; Treatment primary effect: $F_{(1,8)}$= 5.82, *p*=0.034; Treatment/pretreatment interaction: $F_{(1,8)}$= 0.01, *p*= 0.931]. Female: $F_{(3,8)}$= 22.61, *p*<0.001; Pretreatment primary effect: $F_{(1,8)}$= 58.99, *p*<0.001; Treatment primary effect: $F_{(1,8)}$= 7.98, *p*=0.022; Treatment/pretreatment interaction: $F_{(1,8)}$= 0.85, *p*= 0.384.

Figure 8. Male; $F_{(3,8)}$= 1.02, *p*=0.423; Pretreatment chief effect: $F_{(1,8)}$= 0.01, *p*=0.907; Treatment chief effect: $F_{(1,8)}$= 2.56, *p*=0.138; Treatment/pretreatment interaction: $F_{(1,8)}$= 0.09, *p*=0.769). Female: $F_{(3,8)}$= 29.99, *p*<0.001; Pretreatment chief effect: $F_{(1,8)}$= 84.58, *p*<0.001; Treatment chief effect: $F_{(1,8)}$= 0.30, *p*=0.601; Treatment/pretreatment interaction: $F_{(1,8)}$= 5.86, *p*=0.054).

Figure 9A. $F_{(7,16)}$= 19.93, *p*<0.001; Sex principal effect: $F_{(1,16)}$= 1.37, *p*=0.260; Pretreatment principal effect: $F_{(1,16)}$= 50.01, *p*<0.001; Treatment principle effect: $F_{(1,16)}$= 3.96, *p*= 0.064; Treatment/sex interaction: $F_{(1,16)}$= 73.04, *p*< 0.001; Pretreatment/ sex interaction; $F_{(1,16)}$= 0.545, *p*= 0.471; Pretreatment/treatment interaction $F_{(1,16)}$= 7.06, *p*= 0.017; Pretreatment/treatment/sex interaction: $F_{(1,16)}$= 3.53, *p*= 0.079.

Figure 9B. $F_{(7,16)}$= 18.81, *p*<0.001; Sex chief effect: $F_{(1,16)}$= 18.49, *p*<0.001; Pretreatment chief effect: $F_{(1,16)}$= 41.36, *p*<0.001; Treatment chief effect: $F_{(1,16)}$= 2.02, *p*= 0.174; Treatment/sex interaction: $F_{(1,16)}$= 5.45, *p*= 0.033; Pretreatment/sex interaction; $F_{(1,16)}$= 7.05, *p*= 0.017; Pretreatment/treatment interaction $F_{(1,16)}$= 16.75, *p*< 0.001; Pretreatment/treatment/sex interaction: : $F_{(1,16)}$= 40.58, *p*< 0.001.
